# Fragmentation Pathway of Organophosphorus Flame Retardants by Liquid Chromatography–Orbitrap-Based High-Resolution Mass Spectrometry

**DOI:** 10.3390/molecules29030680

**Published:** 2024-02-01

**Authors:** Kangcong Li, Yan Gao, Xiuqin Li, Yan Zhang, Benfeng Zhu, Qinghe Zhang

**Affiliations:** 1 Division of Chemical Metrology and Analytical Science, National Institute of Metrology, Beijing 100029, China; 13097513253@163.com (K.L.); gaoyan@nim.ac.cn (Y.G.); yanzhang_larissa@163.com (Y.Z.); 2 College of Materials and Chemistry, China Jiliang University, Hangzhou 310018, China; zhubenfeng88@cjlu.edu.cn; 3 Key Laboratory of Chemical Metrology and Applications on Nutrition and Health for State Market Regulation, Beijing 100029, China; 4 China Key Laboratory of Groundwater Conservation of MWR, China University of Geosciences, Beijing 100083, China

**Keywords:** organophosphorus flame retardant, fragmentation pathway, liquid chromatography, high resolution mass spectrometry

## Abstract

Organophosphorus flame retardants (OPFRs) have been widely used in polymeric materials owing to their flame retardant and plasticizing effects. Investigating the fragmentation pathway of OPFRs is of great necessity for further discovering and identifying novel pollutants using orbitrap-based high-resolution mass spectrometry (HRMS). A total of 25 OPFRs, including alkyl, halogenated, and aromatic types, were analyzed in this study. The fragmentation pathways of the OPFRs were investigated using orbitrap-based HRMS with high-energy collision dissociation (HCD) in positive mode. The major fragmentation pathways for the three types of OPFRs are greatly affected by the substituents. In detail, the alkyl and halogenated OPFRs underwent three McLafferty hydrogen rearrangements, wherein the substituents were gradually cleaved to form the structurally stable [H_4_PO_4_]^+^ (*m*/*z* = 98.9845) ions. In contrast, the aromatic OPFRs would cleave not only the C-O bond but also the P-O bond, depending on the substituents, to form fragment ions such as [C_6_H_7_O]^+^ (*m*/*z* = 95.0495) or [C_7_H_7_]^+^ (*m*/*z* = 91.0530), among others. Using HRMS improved the accuracy of fragment ion identification, and the pathway became more evident. These fragmentation laws can provide identification information in pollutant screening work and theoretical references for the structural characterization of compounds with diverse substituent structures.

## 1. Introduction

Organophosphorus flame retardants (OPFRs) are common polymer additives employed for flame retardation and plasticization [1,2,3]. In recent years, OPFRs and related degradation products and analogues have been commonly found in water [4], dust [5], sludge [6], and various foods [7,8,9,10,11], and are distributed worldwide [12,13,14]. Investigations have demonstrated that certain OPFRs possess neurotoxic and carcinogenic properties, which can substantially reduce human immunity [15,16]. As a result, OPFRs are gaining increasing attention as potential health hazards.

At present, the detection of OPFRs mainly relies on gas or liquid chromatography-mass spectrometry techniques, wherein the qualitative and quantitative analysis of a limited range of targets can be carried out with reference materials [17,18,19]. However, with thousands of OPFRs and derivatives currently available and continuing to expand, it is crucial to perform both suspect and non-target screening, while the requirements for mass spectrometry information related to OPFRs are becoming increasingly stringent. There are many types of organophosphorus flame retardants (OPFRs), mainly including organophosphate esters, organophosphonates, organophosphate salts, phosphine oxides, and organophosphate heterocycles compounds. Organophosphate esters with P(=O)(OR)3 as their characteristic structure account for 84.2% of the available OPFRs monomers [20]. These compounds may produce the same characteristic fragment ions during mass spectrometry cleavage because they contain the same phosphate skeleton, and these characteristic ions can be used as a basis for non-target screening of organophosphorus compounds. There are some variations in the type and abundance of fragment ions produced by different types of organophosphorus compounds, and these differential features are a tool for further differentiation and identification of the target. According to the substituents, OPFRs are currently classified as alkyl, halogenated, and aromatic [21]: alkyl OPFRs substituents are mainly straight or branched composed of C and H elements, halogenated OPFRs substituents are usually H substituted by chlorine or bromine on alkane chains, and aromatic OPFRs substituents generally contain one or more aromatic rings. Lesage et al. [22] used a deuterium label to study the fragmentation of tri-*n*-butyl phosphate. Deuterium labeling makes the fragmentation mechanism more obvious. However, it is still difficult to obtain relevant deuterium-labeled products for the increasing variety of organophosphorus flame retardants. Ma et al. [23] used gas chromatography tandem mass spectrometry to explain the common fragmentation pathways of 13 OPFRs under electron ionization (EI) sources. They found that alkyl OPFRs follow a rearrangement reaction from the precursor [M]^+^ to [M − R + 2H]^+^, [M − 2R + 3H]^+^, and [M − 3R + 4H]^+^ patterns. Yang et al. [24] optimized the ionization energy of the EI source and summarized the fragmentation pathways and characteristic fragment ion database of 17 OPFRs, which provided a basis for determining OPFRs in complex matrix samples. The high-resolution mass spectrometry (HRMS) screening technique can provide higher index parameters in terms of screening accuracy and sensitivity, improve the accuracy of fragment ion identification, effectively perform high-throughput detection, and reduce false positive and false negative rates. Ye et al. [25] used liquid chromatography tandem HRMS with an atmospheric pressure chemical ionization (APCI) source for target, suspect, and non-target screening of OPFRs in Taihu sediments, using characteristic fragment ions for qualitative analysis of emerging OPFRs, which significantly simplifies the information processing during non-target screening but also requires the researcher to learn the target well enough to remove interferences during subsequent analysis effectively. Since there are many different types of OPFRs substituents and the physicochemical properties of the various OPFRs vary widely [24], the study of the fragmentation pathways of the target compounds and the summary of the relevant mass spectrometry information are a crucial step before the screening process.

This study aims to analyze the main characteristic fragment ions generated during the fragmentation of OPFRs by LC-Orbitrap-HRMS technology using an electrospray ionization (ESI) source. The selected OPFRs include alkyl, halogenated, and aromatic classes, which are widely representative considering the chain length and substituent structure. The fragmentation laws were investigated, and the effective information was applied to the screening of OPFRs in rice samples to provide a basis for detecting and accurately characterizing the targets.

## 2. Results and Discussion

### 2.1. Fragmentation Pathway and Characteristic Ions of Three Types of OPFRs

To analyze the fragmentation pathway of the three types of OPFRs, each compound was injected separately at 200 µg/kg, and precursor ions were fragmented under the step- collision energy by HCD.

#### 2.1.1. Fragmentation Pathway and Characteristic Ions of Alkyl OPFRs

In this study, nine representative alkyl OPFRs were selected (Table 1), including straight chain substituents (TMP, TEP, TnPP, TnBP, TPeP, TBOEP) and branched substituents (TiPP, TiBP, TEHP), corresponding to the substituent groups in the order of methyl, ethyl, *n*-propyl, *n*-butyl, *n*-pentyl, 2-butoxyethyl, isopropyl, isobutyl, and 2-ethylhexyl. Among them, TiPP and TnPP, TiBP and TnBP are two pairs of isomers. 

**Figure 1:**
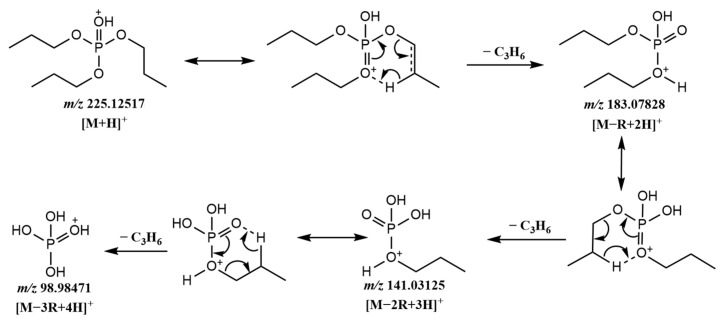
Possible fragmentation pathway of TnPP.

**Figure 2:**
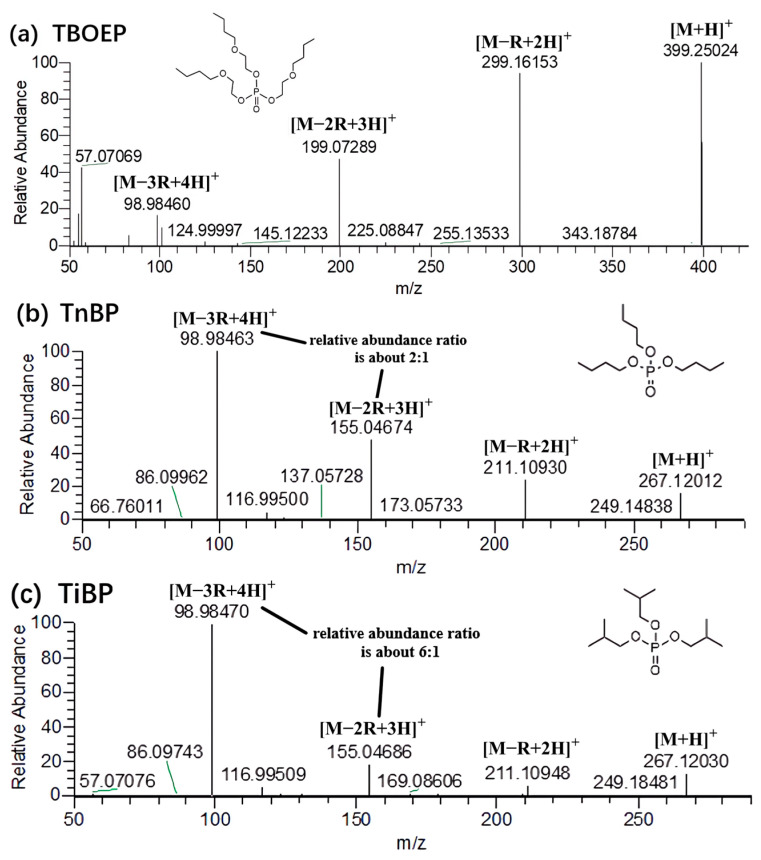

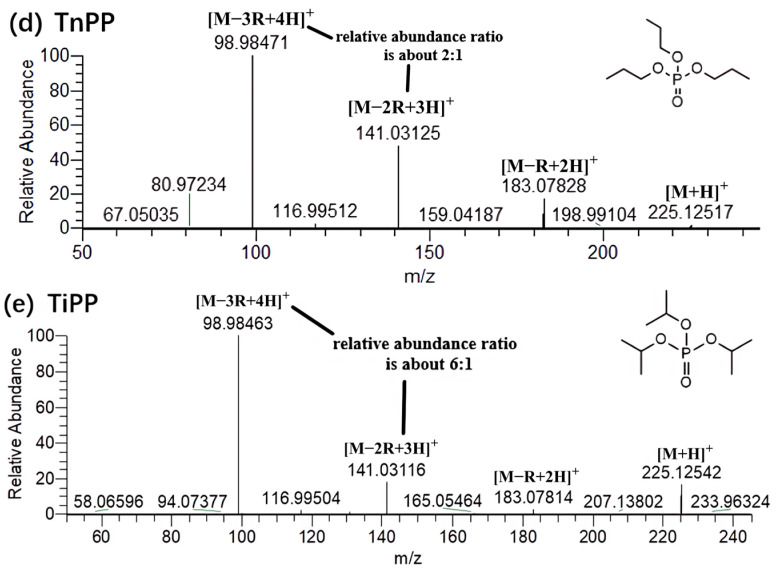
MS^2^ spectra of 5 alkyl OPFR compounds.

In general, alkyl OPFRs underwent three successive McLafferty hydrogen rearrangements within the HCD collision cell. [M − R + 2H]^+^, [M − 2R + 3H]^+^ and [M − 3R + 4H]^+^ ions were produced through repeated rearrangements. Figure 1 shows the possible fragmentation pathway of TnPP. Three rearrangements occurred throughout the process, with each cleavage more likely to occur at the lower bond energy C-O bond (about 326 kJ/mol) compared to the P-O bond (about 410 kJ/mol). The fragmentation process sequentially produced the [C_6_H_16_PO_4_]^+^ (*m*/*z* = 183.0783), [C_3_H_10_PO_4_]^+^ (*m*/*z* = 141.0313), and [H_4_PO_4_]^+^ (*m*/*z* = 98.9847) ions. The abundance ratio and accurate mass of fragment ions were effective bases for qualitative analysis. Among the alkyl OPFRs, most of the compounds had [H_4_PO_4_]^+^ (*m*/*z* = 98.9847) as the base peak, which was a common characteristic fragment ion of alkyl OPFRs. It was very stable in the mass spectrum and could be used as an identifying feature for alkyl OPFRs during screening. However, for TBOEP (Figure 2a), the precursor ion [M + H]^+^ was the base peak. This may be due to the fact that the substituent group of TBOEP was 2-butoxyethyl, and the TBOEP precursor ion was stabilized by the presence of oxygen in the side chain, which led to difficulty in McLafferty hydrogen rearrangement. This feature, in combination with the abundance of fragment ions, could be a basis for screening TBOEP. High-resolution mass spectrometry can provide the accurate mass of the characteristic fragment ions, which provides a guarantee for eliminating interferences and accurately matching molecular formulae during the screening process.

Isomers are difficult to distinguish in qualitative analysis due to their similar structures and same molecular weights but some isomers differ in the type or abundance of fragment ions, which can be used as tools for further identification. Among the alkyl OPFRs, TnBP and TiBP, TnPP and TiPP are two pairs of isomers (Figure 2b–e), in which TiBP and TiPP are branched compounds, and TnBP and TnPP are straight-chain compounds. A comparison of their mass spectra revealed a higher relative abundance ratio of [M − 3R + 4H]^+^ to [M − 2R + 3H]^+^ fragments for the branched compounds and a lower ratio for the straight-chain compounds. For example, the relative abundance ratio of the fragments [M − 3R + 4H]^+^ (*m*/*z* = 98.9846) to [M − 2R + 3H]^+^ (*m*/*z* = 141.0312) of the branched compound TiPP was about 6:1, while the fragments [M − 3R + 4H]^+^ (*m*/*z* = 98.9847) to [M − 2R + 3H]^+^ (*m*/*z* = 141.0313) of the straight-chain compound TnPP was about 2:1. This may be because branched alkanes are more stable than straight-chain alkanes, and when used as substituents, the rearrangement reaction of branched OPFRs mainly occurs in the oxygen atoms connecting the substituent groups, while the energy of straight-chain compounds is dispersed during fragmentation, so the branched compounds are more prone to the formation of [M − 3R + 4H]^+^, and the proportion of the fragments produced is relatively higher. This conclusion can provide a reference for distinguishing isomers during the qualification of compounds.

#### 2.1.2. Fragmentation Pathway and Characteristic Ions of Halogenated OPFRs

In this study, six representative halogenated OPFRs were selected (Table 1), including five chlorinated OPFRs (TCEP, TCIPP, TCPP, T23DCIPP, and TDCIPP) and one brominated OPFR (T23DBPP). The substituents are chloroethyl, 2-chloroisopropyl, chloropropyl, 2,3-dichloropropyl, 1,3-dichloropropyl, and 2,3-dibromopropyl, in which TCIPP and TCPP, T23DCIPP and TDCIPP are two pairs of isomers. 

Due to the high natural abundance of chlorine and bromine isotopes, halogenated OPFRs produce unique ion clusters during ionization. According to the relative abundance ratios of the isotope peaks in the MS^1^ spectrum (Figure S5), the type and amount of halogens can be deduced, which facilitates the characterization of the compounds. The two halogenated OPFRs, TDCIPP and T23DBPP, both had distinctive isotope peaks in the MS^1^ spectrum. For TDCIPP, the relative abundance ratio of the isotope peaks [M + H]^+^:[M + 2 + H]^+^:[M + 4 + H]^+^:[M + 6 + H]^+^:[M + 8 + H]^+^:[M + 10 + H]^+^ was 51.63:100.00:81.46:34.68:7.53:1.06; For T23DBPP, the relative abundance ratio of the isotope peaks [M + H]^+^:[M + 2 + H]^+^:[M + 4 + H]^+^:[M + 6 + H]^+^:[M + 8 + H]^+^:[M + 10 + H]^+^:[M + 12 + H]^+^ was 5.53:31.95:74.54:100.00:73.73:27.96:4.06. The natural abundance ratio of ^35^Cl and ^37^Cl is about 75.77%:24.23%, and the natural abundance ratio of ^79^Br and ^81^Br is about 50.60%:49.31%. After calculation, when a compound contains six Cl elements, the theoretical relative abundance ratio of the isotope peaks [M + H]^+^:[M + 2 + H]^+^:[M + 4 + H]^+^:[M + 6 + H]^+^:[M + 8 + H]^+^:[M + 10 + H]^+^ is 50.00:100.00:83.33:37.04:9.26:1.23, which is essentially identical to the isotope peaks generated by TDCIPP in the experiment. When a compound contains six Br elements, the theoretical relative abundance ratio [M + H]^+^:[M + 2 + H]^+^:[M + 4 + H]^+^:[M + 6 + H]^+^:[M + 8 + H]^+^:[M + 10 + H]^+^:[M + 12 + H]^+^ of the isotope peaks is 5.00:30.00:75.00:100.00:75.00:30.00:5.00. This also matches the experimental isotopic peaks produced by T23DBPP. In the MS^1^ spectrum of TDCIPP, the highest isotopic abundance was observed for the molecular formula C_9_H_15_Cl^37^Cl^35^_5_PO_4_ ([M + 2 + H]^+^ *m*/*z* = 430.8880), and multiple [M + Na]^+^ peaks were also observed. In the MS^1^ spectrum of T23DBPP, the highest isotopic abundance of the molecular formula C_9_H_15_Br^81^_3_Br^79^_3_PO_4_ ([M + 6 + H]^+^ *m*/*z* = 698.5812) was observed along with numerous [M + NH_4_]^+^ peaks.

**Figure 3:**
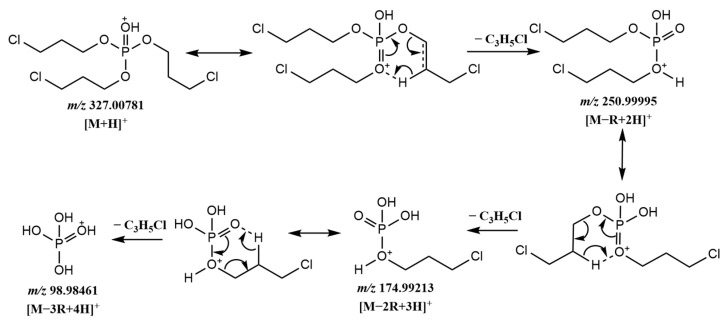
Possible fragmentation pathway of TCPP.

**Figure 4:**
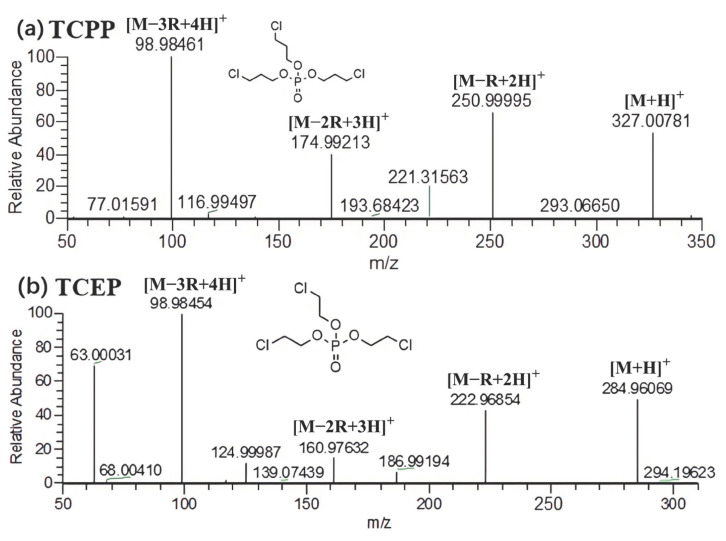
MS^2^ spectra of two halogenated OPFR compounds.

It was found that the fragmentation process of halogenated OPFRs was similar to alkyl OPFRs. The compounds were also gradually fragmented through the McLafferty hydrogen rearrangement reaction to form [M − R + 2H]^+^, [M − 2R + 3H]^+^, and [M − 3R + 4H]^+^ fragment ions, finally generating [H_4_PO_4_]^+^ (*m*/*z* = 98.9845). Taking TCPP as an example, the possible fragmentation pathway of TCPP is shown in Figure 3 and Figure 4a. The whole fragmentation process also involved three rearrangements. During the fragmentation process, the chloropropyl group was fragmented and combined with the surrounding hydrogen to form [C_6_H_14_Cl_2_PO_4_]^+^ (*m*/*z* = 250.9999), [C_3_H_9_ClPO_4_]^+^ (*m*/*z* = 174.9921), and [H_4_PO_4_]^+^ (*m*/*z* = 98.9846) ions. Unlike the alkyl OPFRs, the [M + H]^+^ and [M − R + 2H]^+^ ions were often more prominent in the spectrum. This suggested that halogenated OPFRs were more stable than alkyl OPFRs and less likely to be destroyed at the same collision energy. This may be because the bond length of the C–Cl bond (about 177 × 10^−12^ m) is longer, making it easier to cleave than the C–O bond (about 143 × 10^−12^ m). Among the characteristic fragment ions of some halogenated OPFRs, [M − Cl + H]^+^, [M − R − Cl + 2H]^+^, and [M − 2R − Cl + 3H]^+^ ions are included. Taking TCEP as an example (Figure 4b), except for the common ions [M − R + 2H]^+^ (*m*/*z* = 222.9685), [M − 2R + 3H]^+^ (*m*/*z* = 160.9763), and [M − 3R + 4H]^+^ (*m*/*z* = 98.9845), it also appeared that the loss of chlorine led to the formation of [M − R − Cl + 2H]^+^ (*m*/*z* = 186.9919) and [M − 2R − Cl + 3H]^+^ (*m*/*z* = 124.9999) ions.

#### 2.1.3. Fragmentation Pathway and Characteristic Ions of Aromatic OPFRs

The structures of aromatic OPFRs usually contain one or more aromatic rings. Different from the above-mentioned two types of OPFRs, the substituent types of aromatic OPFRs are more abundant. The experiment involved dBPhP and BdPhP with a benzene ring and n-butyl; EHDPP with a benzene ring and 2-ethylhexyl; IDDP with isodecyl; CDPP with benzene and toluene; TPHP with a benzene ring; T35DMPP with xylene; and isomers *o*-TCP, *m*-TCP, and *p*-TCP with toluene.

**Figure 5:**
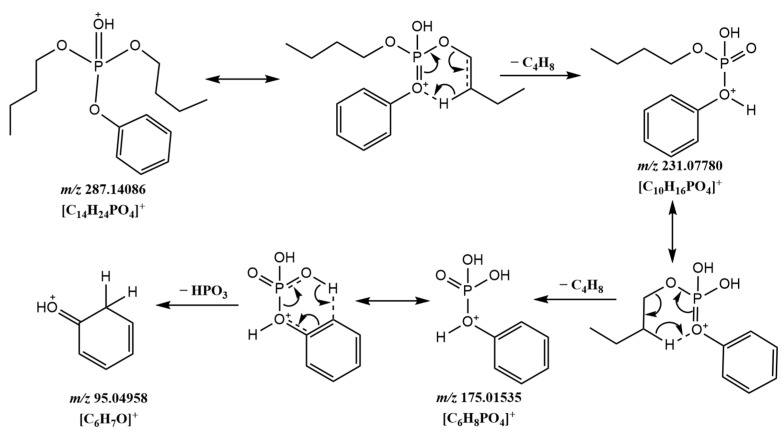
Possible fragmentation pathway of dBPhP.

**Figure 6:**
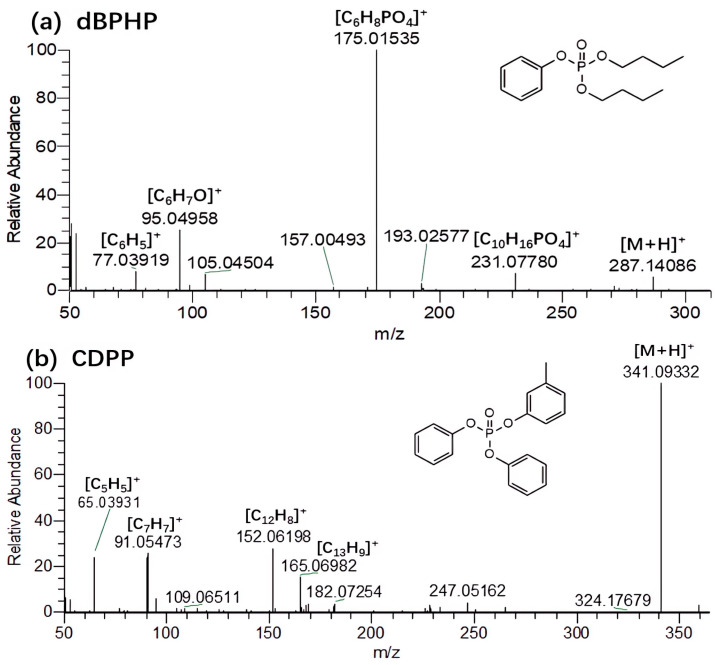
MS^2^ spectra of two aromatic OPFR compounds.

Experiments have shown that the aromatic ring had an influence on the fragmentation of the compound, and its fragmentation pathways and characteristic fragments were different from those of alkyl and halogenated OPFRs. For aromatic OPFRs containing alkane chains in the substituents, such compounds would preferentially cleave the C-O bond connected to the alkane chain during the fragmentation process to form a stable structure. The structure of dBPhP contains one benzene ring and two alkane chains, and the possible fragmentation process and MS^2^ spectra are shown in Figure 5 and Figure 6a. The characteristic fragment ions [C_10_H_16_PO_4_]^+^ (*m*/*z* = 231.0778) and [C_6_H_8_PO_4_]^+^ (*m*/*z* = 175.0154) were formed by cleavage of the C–O bonds on the two alkane chains, followed by cleavage of the P–O bonds to form the ion [C_6_H_7_O]^+^ (*m*/*z* = 95.0496).

The structures of BdPhP, EHDPP, and IDDP contain two benzene rings and an alkane chain. These compounds formed characteristic fragment ions [C_12_H_12_PO_4_]^+^ (*m*/*z* = 251.0465) with two benzene rings by the cleavage of the C–O bond on the alkane chain, and gradually formed [C_12_H_8_]^+^ (*m*/*z* = 152.0620) ions and [C_6_H_7_O]^+^ (*m*/*z* = 95.0496) ions in the subsequent fragmentation process. It was observed that when the substituent of aromatic OPFRs contained an alkane chain, the alkane chain was very easily cleaved, and the structure with a benzene ring was relatively stable, which could be used as characteristic fragment ions in screening.

For aromatic OPFRs in which the three substituents were all aromatic rings, the compounds had a stable structure due to the conjugation effect. Therefore, the precursor peak was the base peak in the MS^2^ spectrum. If the substituent was a benzene ring(TPHP), [C_12_H_8_]^+^ (*m*/*z* = 152.0620) and [C_6_H_7_O]^+^ (*m*/*z* = 95.0496) characteristic fragment ions would be generated; if the aromatic ring was toluene(*o*-TCP, *m*-TCP, *p*-TCP), [C_13_H_9_]^+^ (*m*/*z* = 165.0698), [C_7_H_7_]^+^ (*m*/*z* = 91.0547) and [C_5_H_5_]^+^ (*m*/*z* = 65.0393) characteristic fragment ions would be generated; for compounds containing both toluene and benzene rings like CDPP (Figure 6b), all five characteristic fragment ions would appear. The substituent of T35DMPP was xylene, and its characteristic fragment ions were [C_16_H_18_PO_3_]^+^ (*m*/*z* = 289.0984), [C_14_H_11_]^+^ (*m*/*z* = 179.0853) and [C_6_H_7_]^+^ (*m*/*z* = 79.0548).

### 2.2. Comparison with Fragmentation Pathways under the EI Source

**Figure 7:**
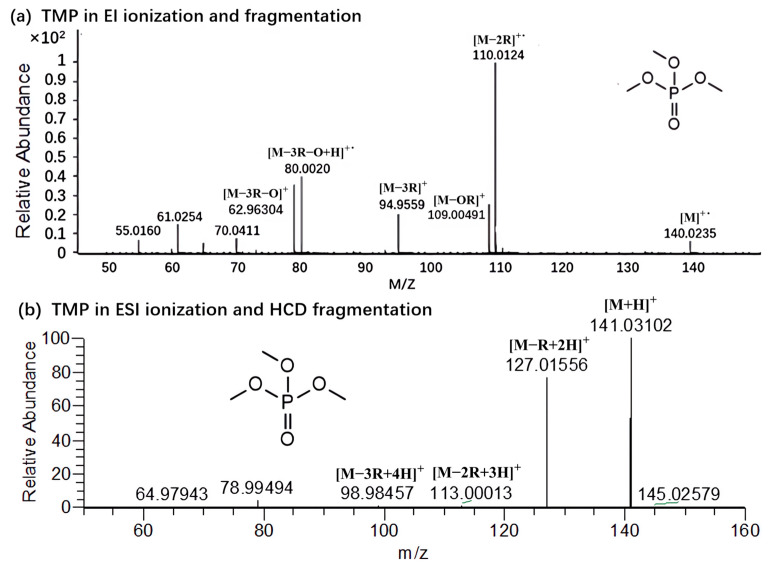
Mass spectra of TMP under two instruments.

The full MS spectra of OPFRs under the EI source are shown in Figure 7a and Figure S6. This paper compared the precursor and fragment ions of OPFRs under the two instruments to study the differences in their ionization and fragmentation characteristics. It was found that after ESI ionization, alkyl and aromatic OPFRs would generate precursors [M + H]^+^, while some halogenated OPFRs would generate [M + NH_4_]^+^ in addition to [M + H]^+^. This may be because the proton affinity of this compound is close to that of ammonium. [M + Na]^+^ was found in the MS^1^ spectrum of most OPFRs, usually due to impurities in the sample vial, liquid phase line, or solvent [26]. The ion clusters characteristic of the halogenated OPFRs made the [M + NH_4_]^+^ and [M + Na]^+^ more prominent. The ionization voltage of the EI source was usually 70 eV, and the precursors were often fragmented by ionization. So, the precursor ion information could not be presented in the mass spectrum. By comparing the fragment ions under the two instruments, it was found that the fragmentation pathways of most alkyl and halogenated OPFRs were similar, and usually underwent McLafferty hydrogen rearrangement after generating precursors. [M − R + 2H]^+^, [M − 2R + 3H]^+^, [M − 3R + 4H]^+^ fragment ions were formed after the chemical bond was cleaved. However, there were still significant differences in the ionization and fragmentation of some compounds between the two instruments, such as TMP (Figure 7), which followed the above rearrangement rules under the ESI ionization and HCD fragmentation, producing [C_2_H_8_PO_4_]^+^ (*m*/*z* = 127.0156), [CH_6_PO_4_]^+^ (*m*/*z* = 113.0001), and [H_4_PO_4_]^+^ (*m*/*z* = 98.9846) fragment ions. While under the EI ionization and fragmentation, TMP lost the methyl group by the C–O bond cleavage to form [CH_3_PO_4_]^+^ (*m*/*z* = 110.0124) and [PO_4_]^+^ (*m*/*z* 94.9559) fragment ions. For aromatic OPFRs, due to the relatively stable structure, precursor peaks could be observed in the fragmentation spectrum under the EI source. The characteristic fragment ion of OPFRs with a benzene ring as the substituent was [C_6_H_7_O]^+^ (*m*/*z* = 95.0495) under the ESI ionization and HCD fragmentation, and [C_6_H_6_O]^+^ (*m*/*z* = 94.0409) under the EI source; the OPFR compound whose substituent was toluene would produce characteristic fragment ions of [C_7_H_7_]^+^ (*m*/*z* = 91.0530) and [C_5_H_5_]^+^ (*m*/*z* = 65.0393) in both fragmentation modes.

In general, due to the different ionization methods and fragmentation principles, there will be some differences in the fragmentation pathways and fragment ions generated under the two instruments. The fragmentation spectrum of the EI source usually cannot display the precursor ion information, which makes some references missing in the screening process. However, this ion source is easy to use and obtains abundant fragment ions, often used to establish a standard spectrum. Compared with the EI ionization and fragmentation, the ESI ionization and HCD fragmentation have wider applicability. The soft ionization method can retain the information of the precursor ions, acquire the accurate mass of the precursor ions in high-resolution mass spectrometry to achieve the accurate matching of molecular formulae, and combine with the HCD, which can be fragmented at different collision energies, to further confirm the structure of the compounds by fragment ions and provide a complete basis for the screening experiments. Although the ion fragmentation through the collision cell increases the complexity of the experimental operation, it is beneficial for the experimenter to discover more rules by adjusting the parameters. The comparison of the two instruments has greatly contributed to the target mass spectrum database, providing strong support for the screening of new pollutants.

### 2.3. Screening of Organophosphorus Flame Retardants in Actual Samples

Mass spectrometry information that can be used for qualitative analysis was obtained by exploring the fragmentation pathways of organophosphorus compounds under LC-HRMS, and in order to preliminarily verify the suitability of the relevant mass spectrometry information, an experiment was conducted to screen OPFR in rice samples under the same chromatographic and mass spectrometry conditions as those described in Section 3.2 in the text. Firstly, the target chromatographic peaks were extracted from the tested rice samples by the accurate mass of the precursor ions. Then, the extracted precursor ions were sent to the HCD collision cell for fragmentation, and the MS^2^ spectrum was matched to the standard solution spectrum. Compounds with less than two characteristic fragmentation ions were identified as false positives and further analyzed based on the mass spectrometry information generated by fragmentation.

**Figure 8:**
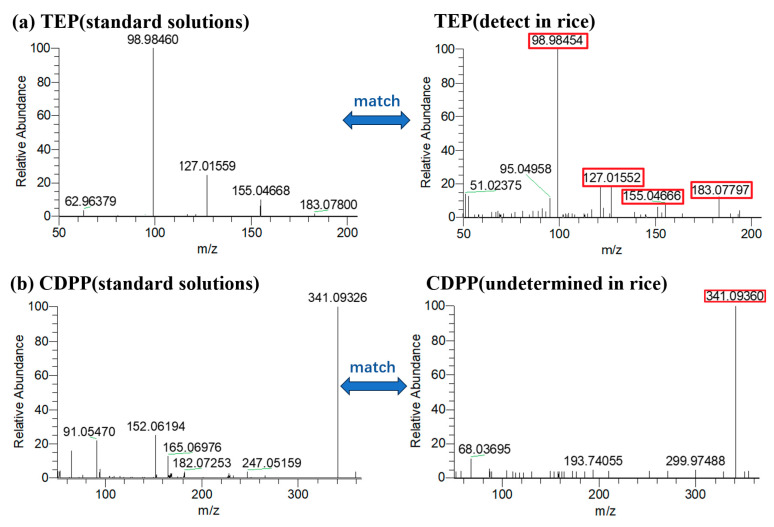
Matching of MS^2^ spectra of (**a**) TEP and (**b**) CDPP in standard solutions and rice samples.

As shown in Figure 8, two undetermined compounds, TEP (*m*/*z* = 183.0779) and CDPP (*m*/*z* = 341.0936), were extracted from rice samples by the accurate mass of precursor ions. The four characteristic ions in the MS^2^ spectrum of TEP are consistent with the standard solution spectrum, and the mass deviation of the characteristic ions is less than 5 ppm, with a relative abundance deviation of less than 20%, indicating the actual detected compound. However, according to the MS^2^ spectrum, the undetermined CDPP cannot generate corresponding fragment ions in HCD, making it difficult to accurately identify. Even with the use of HRMS, screening based solely on the accurate mass of precursor ions may still result in some false positives. In order to make more accurate judgments, it is necessary to study the fragment pathways and fragment ions of compounds.

## 3. Materials and Methods

### 3.1. Main Materials and Reagents

Basic information on OPFRs is provided in Table 1. The structure of these OPFRs is shown in Figure S1 in the Supporting Information (SI). Standards of the 25 OPFRs were purchased from Chemservice (West Chester, PA, USA), Dr. Ehrenstorfer (Ausburg, Germany), BePure (Beijing, China), Sigma-Aldrich (Steinheim, Germany), or TRC Inc. (Ontario, ON, Canada). All solvents were HPLC grade: formic acid was purchased from ThermoFisher Scientific (Waltham, MA, USA), methanol (MeOH) was purchased from Merck (Darmstadt, Germany), ultrapure water was prepared with a Milli-Q system (Millipore, MA, USA). Primary secondary amine (PSA), octadecylsilane (C18), and zirconium dioxide-coated silica (Z-sep) were purchased from Sigma-Aldrich (Darmstadt, Germany). Magnesium sulfate (MgSO_4_), sodium chloride (NaCl), disodium hydrogen citrate (C_6_H_6_Na_2_O_7_), and sodium citrate (C_6_H_5_Na_3_O_7_) were purchased from Agela Technologies (Torrance, CA, USA). Rice samples were collected from various regions in China (Dongbei, Hubei, and Guangxi) and stored in sealed containers at −20 °C until analysis.

### 3.2. Analytical Methods

These compounds were analyzed on Thermo Vanquish-Q Exactive Plus HRMS with an ESI source. Chromatographic separations were achieved on the Thermo Hypersil GOLD column (100 mm × 2.1 mm, 1.9 μm): 2 μL of the solution was injected at a flow rate of 0.25 mL/min and a column temperature of 30 °C. The mobile phase consisted of water (A) and methanol (B) (both with 0.01% formic acid and 5 mM ammonium formate). The LC gradient elution conditions were as follows: 0~1 min (1% B), 1~3 min (1~39% B), 3~14 min (39~99% B), 14~17 min (99% B), 17.1~21 min (1% B). The total run time was 21 min. Data acquisition was performed using the full mass-parallel reaction monitor (PRM) in positive mode with a mass range of *m*/*z* 100–1500. MS^1^ resolution was 70,000, and MS^2^ resolution was 35,000. The step-collision energy (CE) of HCD was set to 10, 40, and 80 eV, and the spray voltage was set to 3 kV. The capillary temperature was 350 °C, the auxiliary gas heater temperature was 300 °C, the automatic gain control of the number of ions entering the orbitrap (AGC target) was 1 × 10^6^, and the maximum injection time was 100 ms.

The analysis of OPFR under EI source was performed by a GC-QTOF/MS system. Details of parameters and MS^2^ spectra are given in Text S1 and Figure S6 of the Supplementary Materials.

### 3.3. Sample Preparation and Pretreatment

Stock standard solutions of OPFRs were prepared in methanol in amber vials, and working standard solutions of lower concentrations were obtained by serial dilution with methanol. All standard solutions were stored at 4 °C.

The rice samples were ground to a powder. Then, 10 mL of an acetonitrile solution containing 0.5% formic acid was added to 1 g of the sample. After that, 4 g of MgSO_4_, 1 g of NaCl, 0.5 g of C_6_H_6_Na_2_O_7_, and 1 g of C_6_H_5_Na_3_O_7_ were added. The mixture was shaken well and ultrasonicated for 5 min in a water bath. The mixture was vortexed for 1 min and centrifuged at 10,000 rpm for 5 min. The supernatant was extracted and transferred to a centrifuge tube containing 50 mg of PSA, 50 mg of C18, and 150 mg of MgSO_4_. The mixture was vortexed for 1 min and centrifuged at 10,000 rpm for 5 min. The supernatant was filtered with a PTFE membrane (0.22 μm) and dried under mild nitrogen. After the solution was reconstituted with 1 mL of methanol and centrifuged at 10,000 rpm for 5 min, 0.8 mL of the supernatant was taken for analysis.

## 4. Conclusions

In this study, the LC-HRMS method was used to investigate the fragmentation laws of 25 OPFR compounds, which were divided into three types: alkyl, halogenated, and aromatic. Each compound’s fragmentation pathways and characteristic fragment ions were analyzed to provide a basis for results processing during the screening process. It was found that the collision stability of alkyl OPFRs was the worst, followed by halogenated OPFRs and aromatic OPFRs. In terms of characteristic fragment ions, both alkyl and halogenated types could form structurally stable [H_4_PO_4_]^+^ (*m*/*z* = 98.9845) ions, but aromatic OPFRs produced characteristic fragment ions such as [C_6_H_8_PO_4_]^+^ (*m*/*z* = 175.0155), [C_12_H_12_PO_4_]^+^ (*m*/*z* = 251.0465), and [C_12_H_8_]^+^ (*m*/*z* = 152.0620) according to different substituents. These characteristic fragment ions provide identification signals in follow-up screening work and theoretical references for the rapid recognition of OPFRs’ targets. Through the analysis of several isomers, it was found that in alkyl OPFR compounds, the relative abundance ratio of [M − 3R + 4H]^+^ to [M − 2R + 3H]^+^ fragment ions produced by compounds with branched substituents was higher, which was about 6:1. In contrast, the ratio of [M − 3R + 4H]^+^ to [M − 2R + 3H]^+^ fragment ions with straight substituents was lower, about 2:1. This ratio can be used as an index to distinguish several kinds of isomers in the subsequent screening process, assist in judging the structural types of substituents, and make more detailed qualitative analysis of the compounds found. The application of OPFRs’ fragmentation laws in food and environmental samples will be further investigated to expand the scope of our research in the future.

## Figures and Tables

**Table 1:** OPFRs’ material information.

Number	Compound	Abbreviation	*M_r_*	Molecular Formula	CAS Number
**1**	Trimethyl phosphate	TMP	140.0233	C_3_H_9_PO_4_	512-56-1
**2**	Triethyl phosphate	TEP	182.0702	C_6_H_15_PO_4_	78-40-0
**3**	Tri-*n*-propyl phosphate	TnPP	224.1177	C_9_H_21_PO_4_	513-08-6
**4**	Tri-*n*-butyl phosphate	TnBP	266.1641	C_12_H_27_PO_4_	126-73-8
**5**	Tri-iso-butyl phosphate	TiBP	266.1641	C_12_H_27_PO_4_	126-71-6
**6**	Tripentyl phosphate	TPeP	308.2111	C_15_H_33_PO_4_	2528-38-3
**7**	Tris(2-methylpropyl) phosphate	TiPP	224.1177	C_9_H_21_PO_4_	513-02-0
**8**	Tris(2-ethylhexyl) phosphate	TEHP	434.3519	C_24_H_51_PO_4_	78-42-2
**9**	Tris(2-butoxyethyl) phosphate	TBOEP	398.2428	C_18_H_39_PO_7_	78-51-3
**10**	Tris(chloroethyl) phosphate	TCEP	283.9533	C_6_H_12_Cl_3_PO_4_	115-96-8
**11**	Tris(chloropropyl) phosphate	TCPP	326.0008	C_9_H_18_Cl_3_PO_4_	1067-98-7
**12**	Tris(2-chloroisopropyl) phosphate	TCIPP	326.0008	C_9_H_18_Cl_3_PO_4_	13674-84-5
**13**	Tris(2,3-dichloro-2-propyl)phosphate	T23DCPP	427.8834	C_9_H_15_Cl_6_PO_4_	78-43-3
**14**	Tris(1,3-dichloro-2-propyl)phosphate	TDCIPP	427.8834	C_9_H_15_Cl_6_PO_4_	13674-87-8
**15**	Tris(2,3-dibromopropyl) phosphate	T23DBPP	691.5803	C_9_H_15_Br_6_PO_4_	126-72-7
**16**	Triphenyl phosphate	TPHP	326.0702	C_18_H_15_PO_4_	115-86-6
**17**	2-Ethylhexyl-diphenyl phosphate	EHDPP	362.1641	C_20_H_27_PO_4_	1241-94-7
**18**	Tris-*o*-tolyl-phosphate	*o*-TCP	368.1172	C_21_H_21_PO_4_	78-30-8
**19**	Tris-*m*-tolyl-phosphate	*m*-TCP	368.1172	C_21_H_21_PO_4_	563-04-2
**20**	Tri-*p*-tolyl-phosphate	*p*-TCP	368.1172	C_21_H_21_PO_4_	78-32-0
**21**	Dibutyl phenyl phosphate	dBPhP	286.1328	C_14_H_23_PO_4_	2528-36-1
**22**	Butyl diphenyl phosphate	BdPhP	306.1015	C_16_H_19_PO_4_	2752-95-6
**23**	Tris(3,5-dimethylphenyl) phosphate	T35DMPP	410.1641	C_24_H_27_PO_4_	25653-16-1
**24**	Cresyl diphenyl phosphate	CDPP	340.0859	C_19_H_17_PO_4_	26444-49-5
**25**	Isodecyl diphenyl phosphate	IDDP	390.1954	C_22_H_31_PO_4_	29761-21-5

**1**–**9** are alkyl OPFRs, **10**–**15** are halogenated OPFRs, **16**–**25** are aromatic OPFRs.

## Data Availability

The data presented in this study are available in article and Supplementary Materials.

## Supplementary Materials

The following supporting information can be downloaded at: https://www.mdpi.com/article/10.3390/molecules29030680/s1, Figure S1: The structure of 25 OPFRs; Figures S2–S4: MS^2^ spectrums of OPFRs under the ESI source; Figure S5: MS^1^ spectra of TDCIPP and T23DBPP; Text S1: Instrument parameter details under the EI source; Figure S6: MS^2^ spectrums of OPFRs under the EI source.
