# Supplementary material for: Fragmentation Pathway of Organophosphorus Flame Retardants by Liquid Chromatography–Orbitrap-Based High-Resolution Mass Spectrometry
Source: Molecules. 2024 Feb 1;29(3):680. doi: 10.3390/molecules29030680 (PMC10856799; doi:10.3390/molecules29030680)
Supplement: Supplementary file 1 [file molecules-29-00680-s001.zip › molecules-2791529-supplementary.pdf]

Supplementary Materials

Fragmentation pathway of organophosphorus flame retardants by liquid chromatography-orbitrap based high resolution mass spectrometry

Kangcong Li[1,3], Yan Gao[1,2], Xiuqin Li[1,2*], Yan Zhang[1,4], Benfeng Zhu[3], Qinghe Zhang[1,2*]

1 Division of Chemical Metrology and Analytical Science, National Institute of Metrology, Beijing 100029, China

2 Key Laboratory of Chemical Metrology and Applications on Nutrition and Health for State Market Regulation, Beijing 100029, China

3 College of Materials and Chemistry, China Jiliang University, Hangzhou 310018, China

4 China Key Laboratory of Groundwater Conservation of MWR, China University of Geosciences, Beijing 100083, China

*Correspondence: lixq@nim.ac.cn (X.L.); zhangqh@nim.ac.cn (Q.Z.); Tel.: +86-10-64524784 (Q.Z.)

Figure S1. The structure of 25 OPFRs; Figure (S2-S4). MS^2 spectrums of OPFRs under ESI source; Figure S5. MS^1 spectrums of TDCIPP and T23DBPP; Text S1. Instrument parameter details under EI source; Figure S6. MS^2 spectrums of OPFRs under EI source

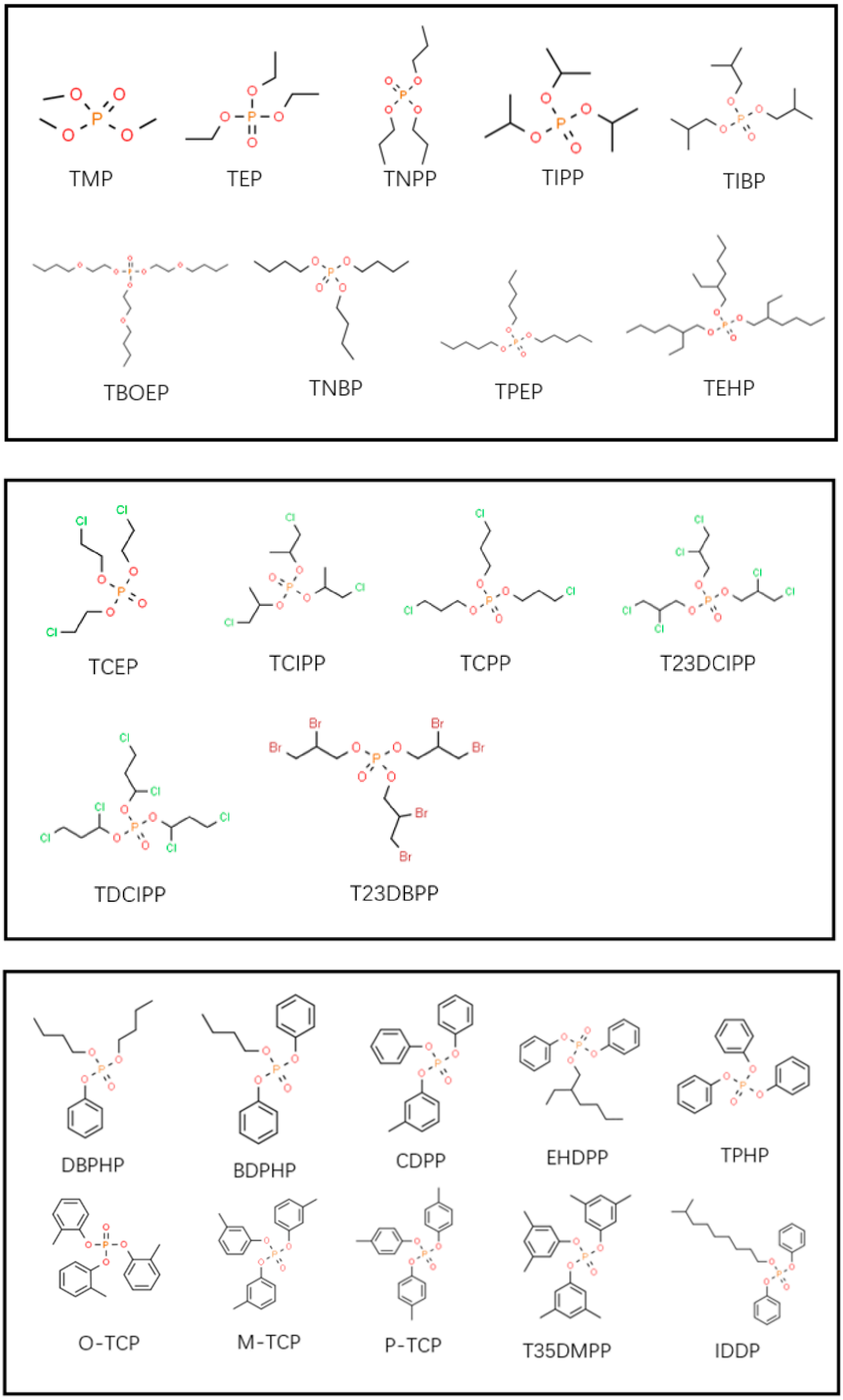

Figure S1. The structure of 25 OPFRs

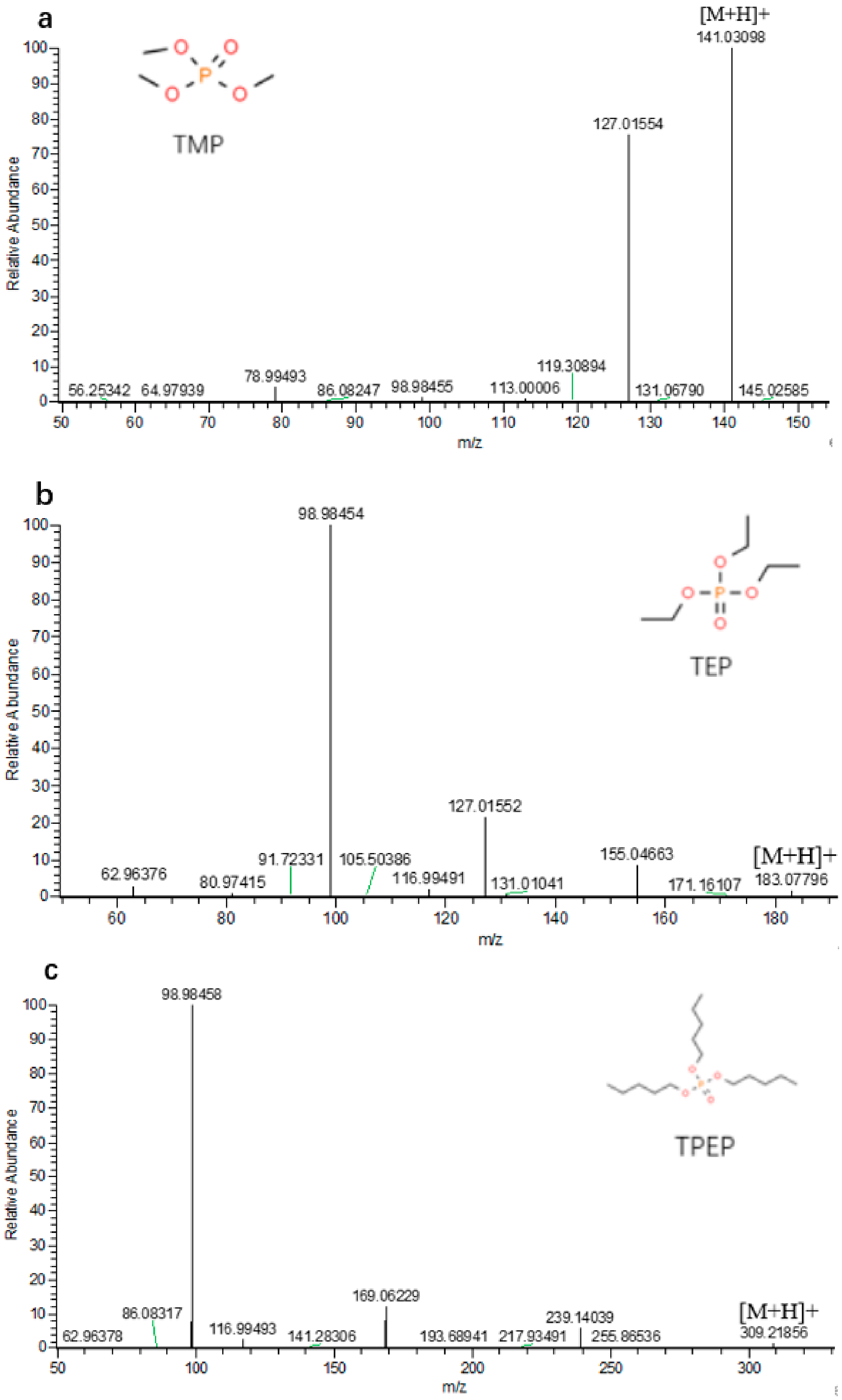
a
TMP
[M+H]+
141.03098
127.01554
119.30894
78.99493
56.25342
64.97939
86.08247
98.98455
113.00006
131.06790
145.02585
Relative Abundance
m/z
b
TEP
98.98454
127.01552
155.04663
[M+H]+
183.07796
62.96376
80.97415
91.72331
105.50386
116.99491
131.01041
171.16107
Relative Abundance
m/z
c
TPEP
98.98458
169.06229
239.14039
[M+H]+
309.21856
62.96378
86.08317
116.99493
141.28306
193.68941
217.93491
255.86536
Relative Abundance
m/z

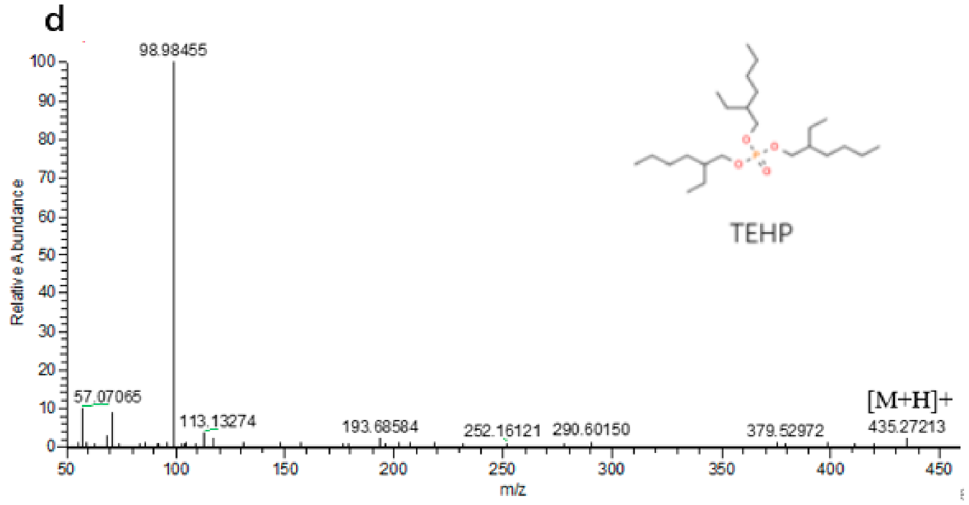

Figure S2. MS² spectrums of 4 alkyl OPFRs under ESI source

a. TMP; b. TEP; c. TPEP; d. TEHP.

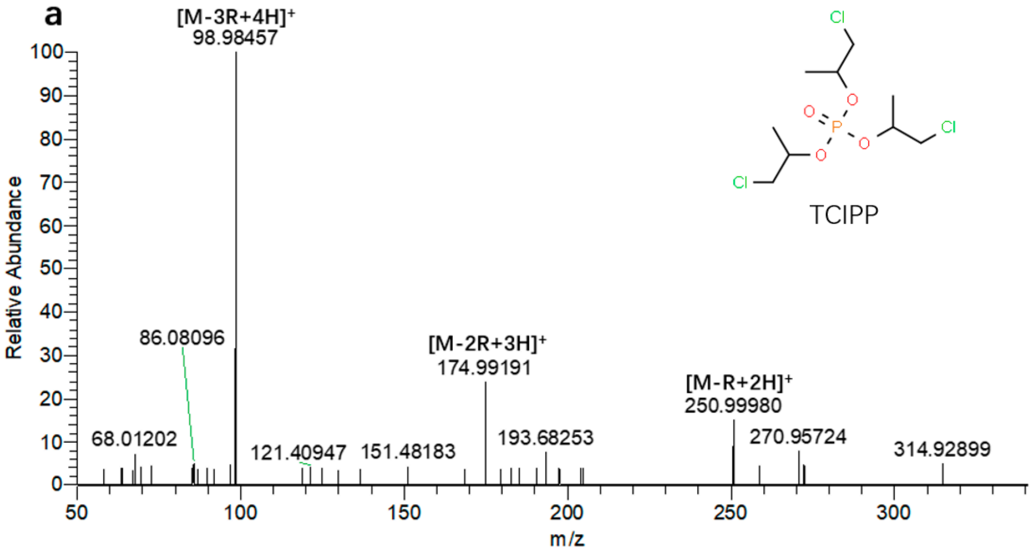

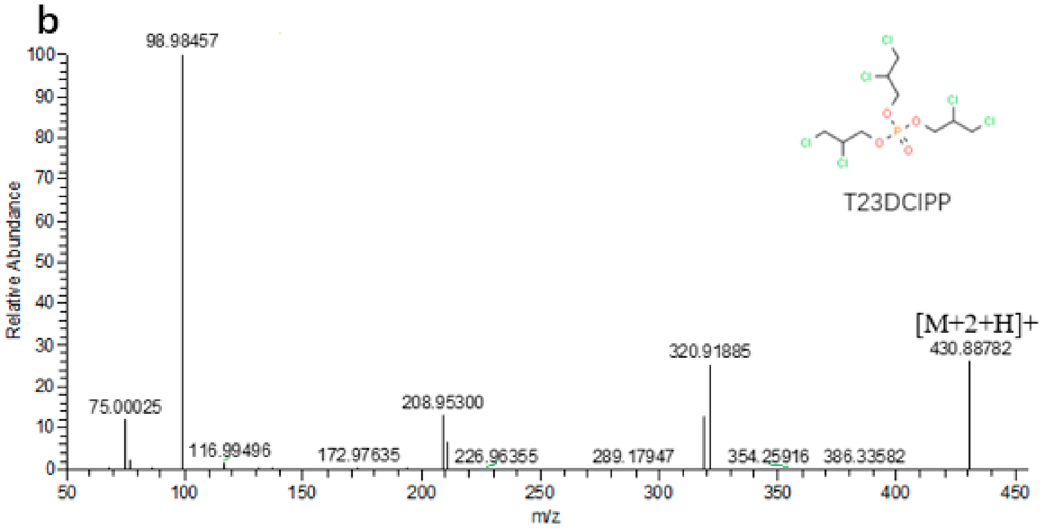

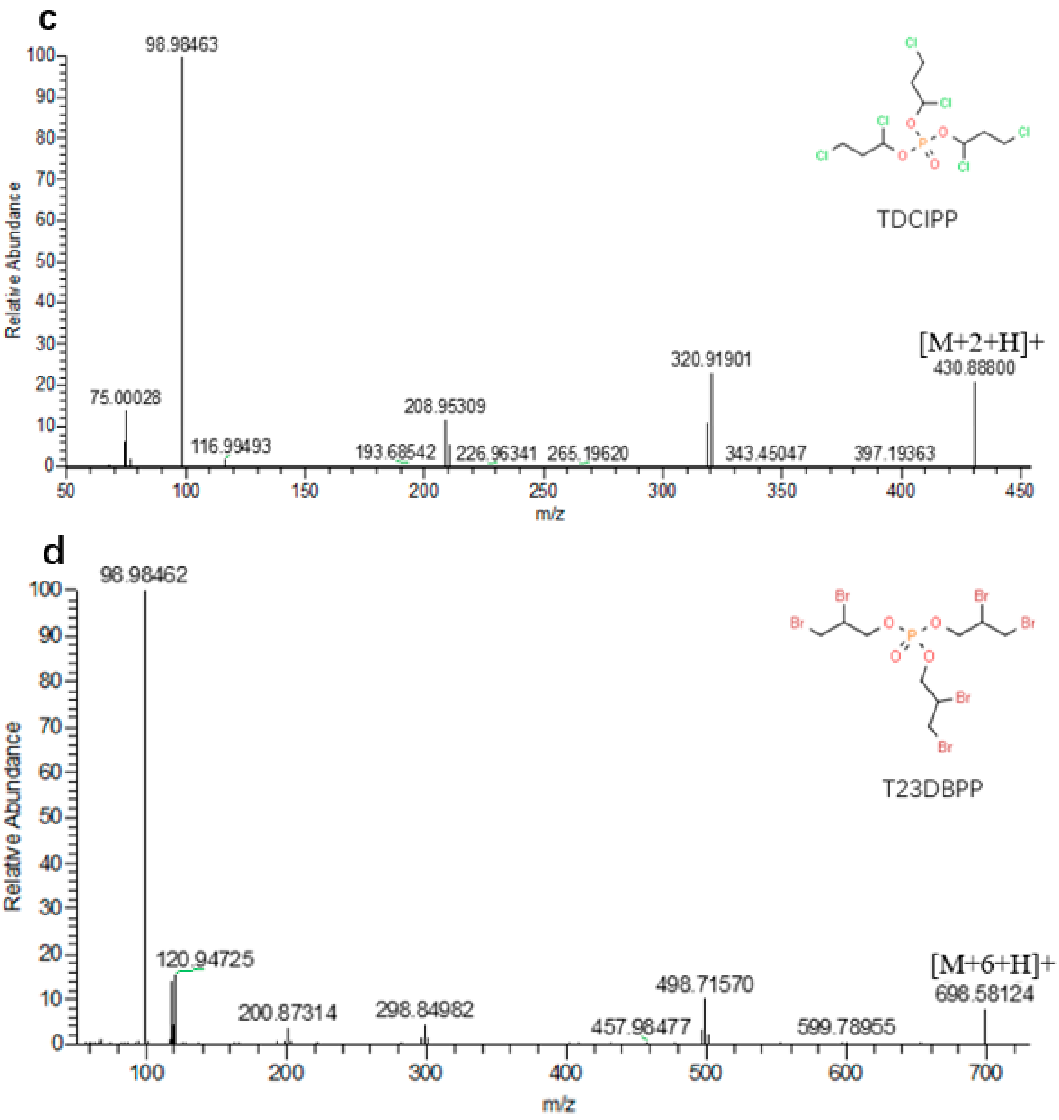

Figure S3. MS^2 spectrums of 4 halogenated OPFRs under ESI source

a. TCIPP; b. T23DCIPP; c. TDCIPP; d. T23DBPP.

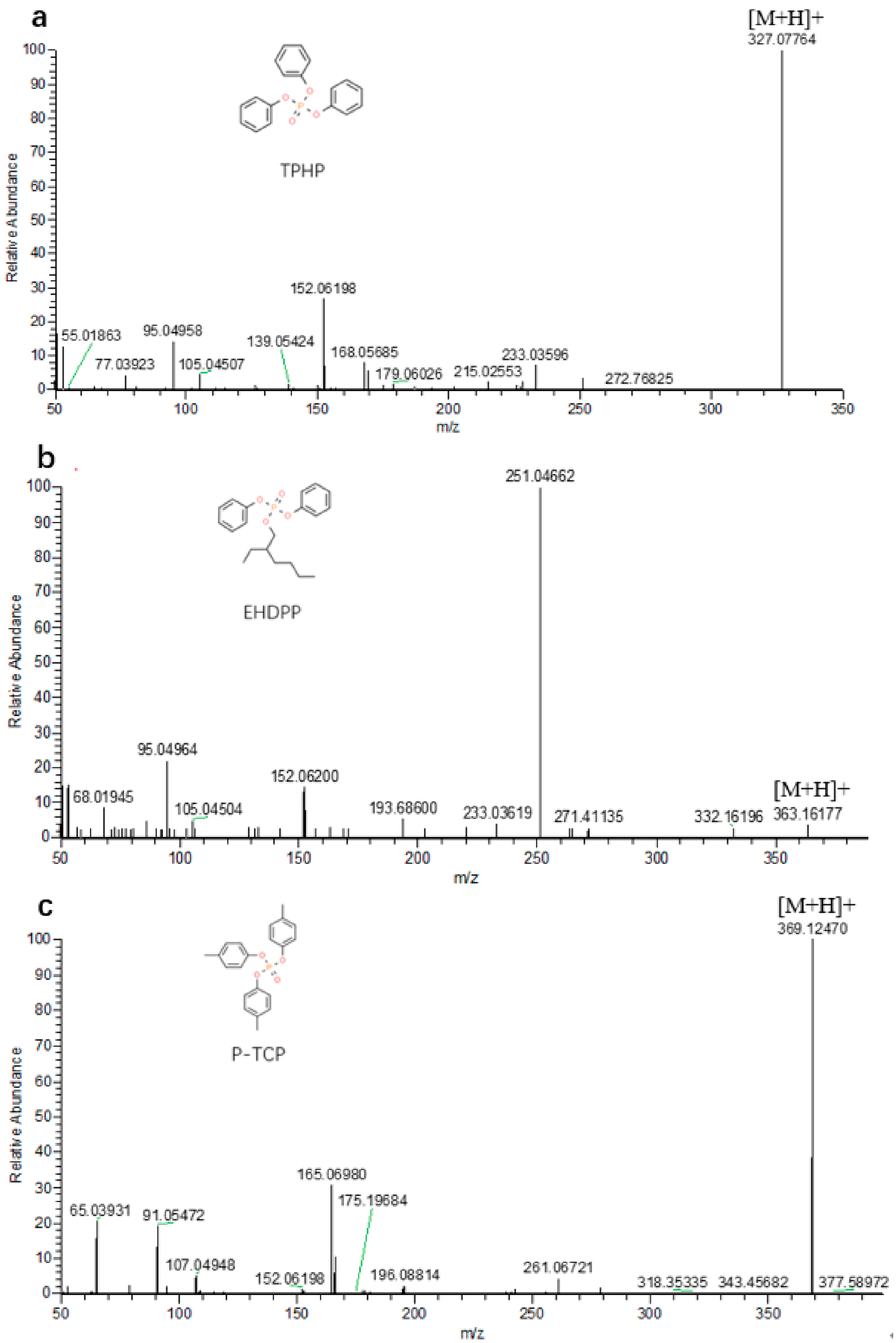
a
[M+H]+
327.07764
TPHP
Relative Abundance
100
90
80
70
60
50
40
30
20
10
0
152.06198
55.01863
95.04958
139.05424
77.03923
105.04507
168.05685
233.03596
179.06026
215.02553
272.76825
50
100
150
200
250
300
350
m/z
b
251.04662
EHDPP
Relative Abundance
95.04964
152.06200
68.01945
105.04504
193.68600
233.03619
271.41135
[M+H]+
332.16196
363.16177
m/z
c
[M+H]+
369.12470
P-TCP
Relative Abundance
165.06980
175.19684
65.03931
91.05472
107.04948
152.06198
196.08814
261.06721
318.35335
343.45682
377.58972
m/z

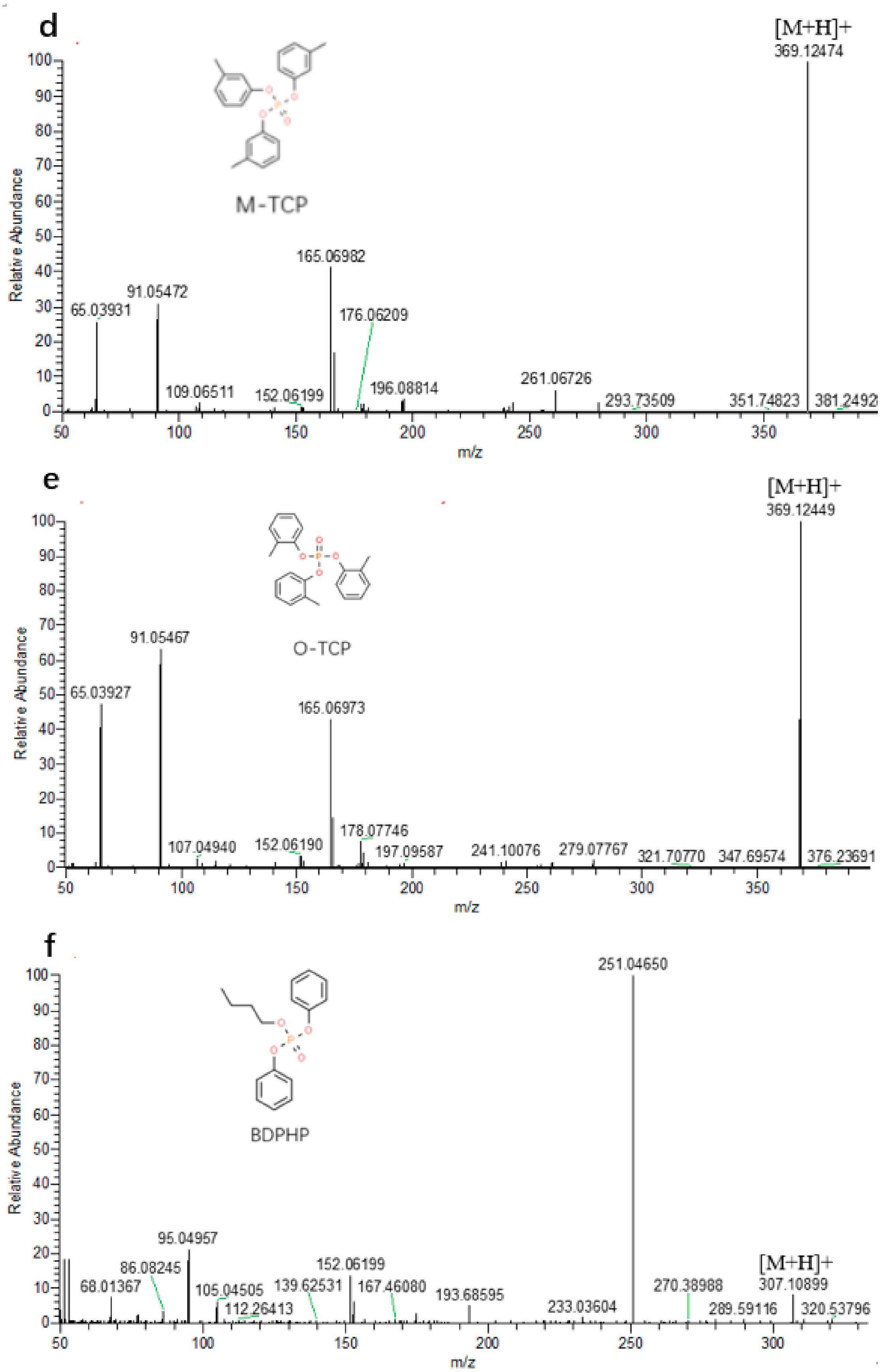
d
[M+H]+
369.12474
M-TCP
Relative Abundance
165.06982
91.05472
65.03931
176.06209
109.06511
152.06199
196.08814
261.06726
293.73509
351.74823
381.2492
m/z
e
[M+H]+
369.12449
O-TCP
Relative Abundance
91.05467
65.03927
165.06973
107.04940
152.06190
178.07746
197.09587
241.10076
279.07767
321.70770
347.69574
376.23691
m/z
f
251.04650
BDPHP
Relative Abundance
95.04957
86.08245
68.01367
105.04505
112.26413
139.62531
152.06199
167.46080
193.68595
233.03604
270.38988
[M+H]+
307.10899
289.59116
320.53796
m/z

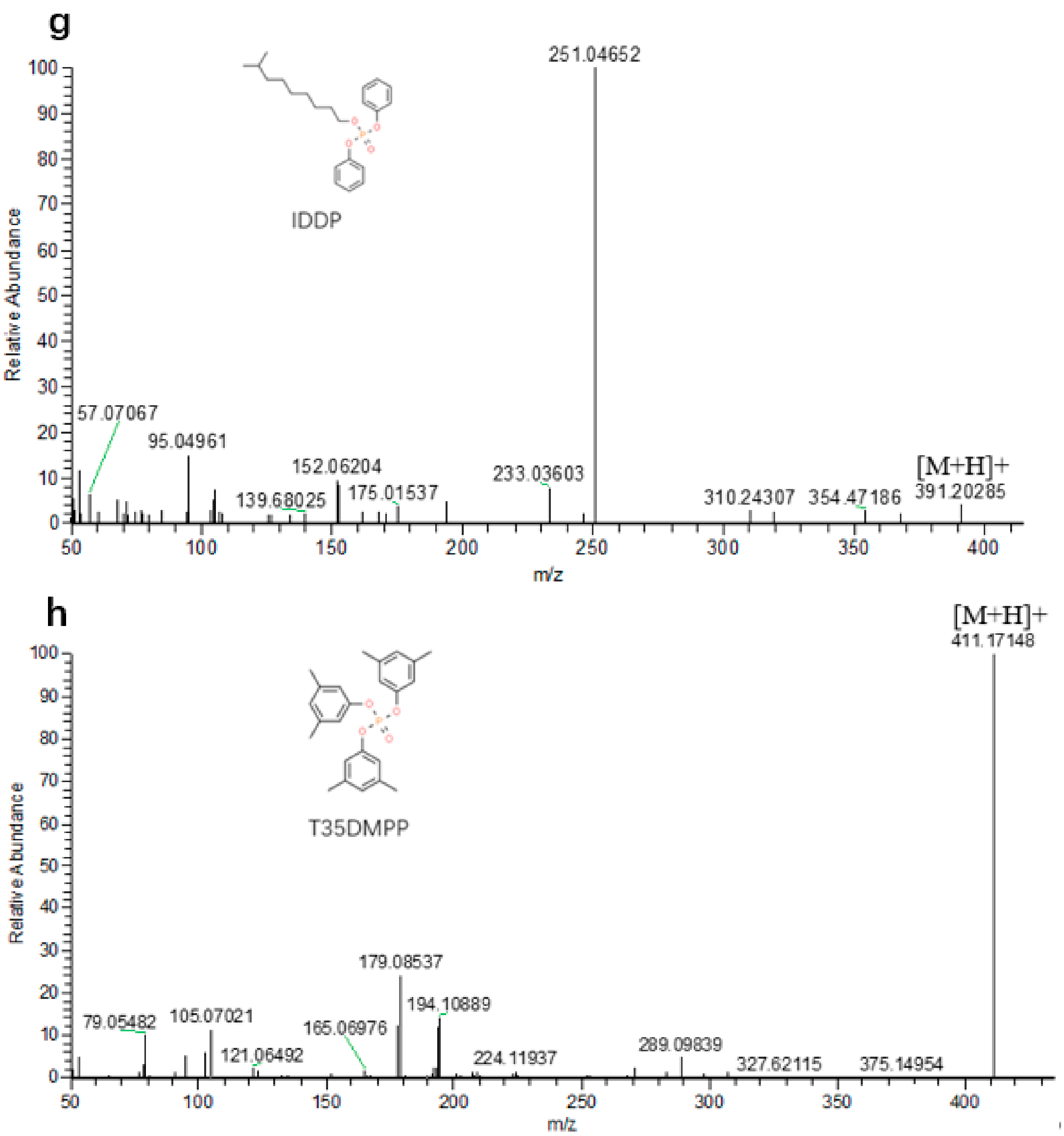

Figure S4. MS^2 spectrums of 8 aromatic OPFRs under ESI source

a. TPHP; b. EHDPP; c. P-TCP; d. M-TCP; e. O-TCP; f. BDPHP; g. IDDP; h. T35DMPP.

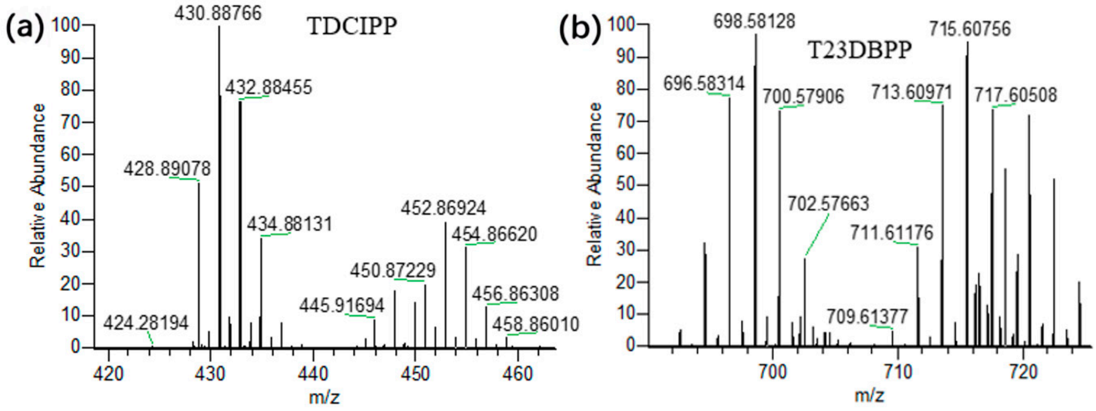

Figure S5. MS^1 spectrums of TDCIPP and T23DBPP

a. TDCIPP; b. T23DBPP

Text S1. Instrument parameter details under EI source

The analysis of OPFR under EI source was performed by a GC-QTOF/MS system consisting of a 7890 B gas chromatography system and a 7200 quadrupole time-of-flight mass spectrometer (Agilent Technologies, Santa Clara, USA). OPFRs were separated on an Agilent DB-5MS-Ultra Inert (30 m × 0.25 mm × 0.25 μm) column. The GC oven temperature was initiated at 50°C and held for 1 min, increased by 20°C /min to 280°C and held for 1 min, then further increased to 300°C by 30°C /min and held for 10 min. The QTOF-MS ion source and quadrupole analyser temperatures were set at 230°C and 150°C, respectively. Mass spectrometric ionization was undertaken in full can mode with an EI voltage of 50 eV, and TOF for MS was operated at 1 spectrum/s, acquiring the mass range m/z 50–450. [1]

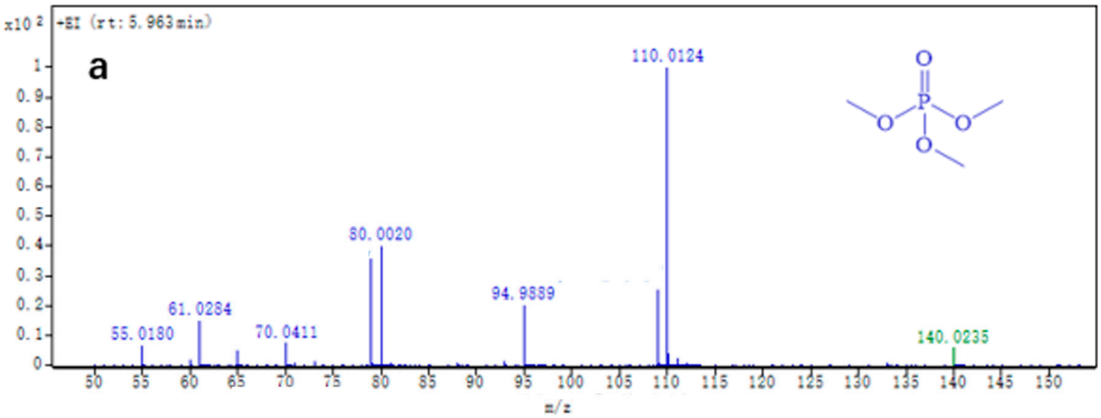

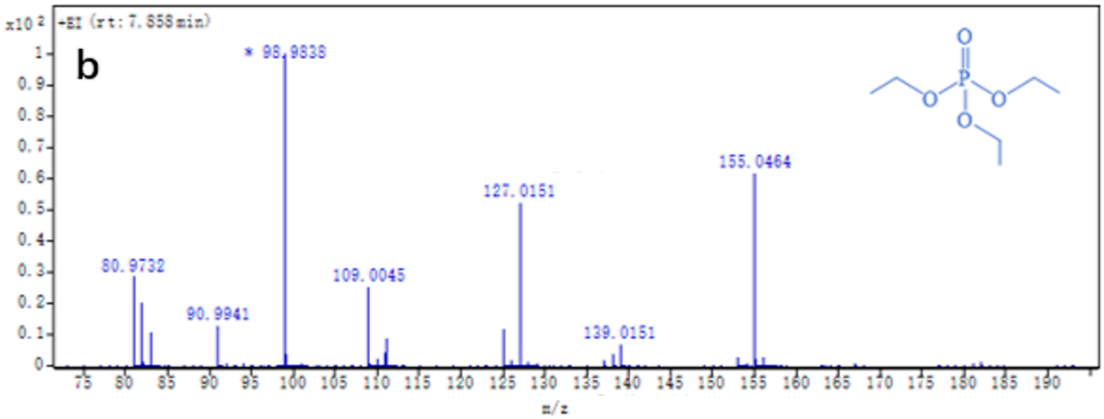

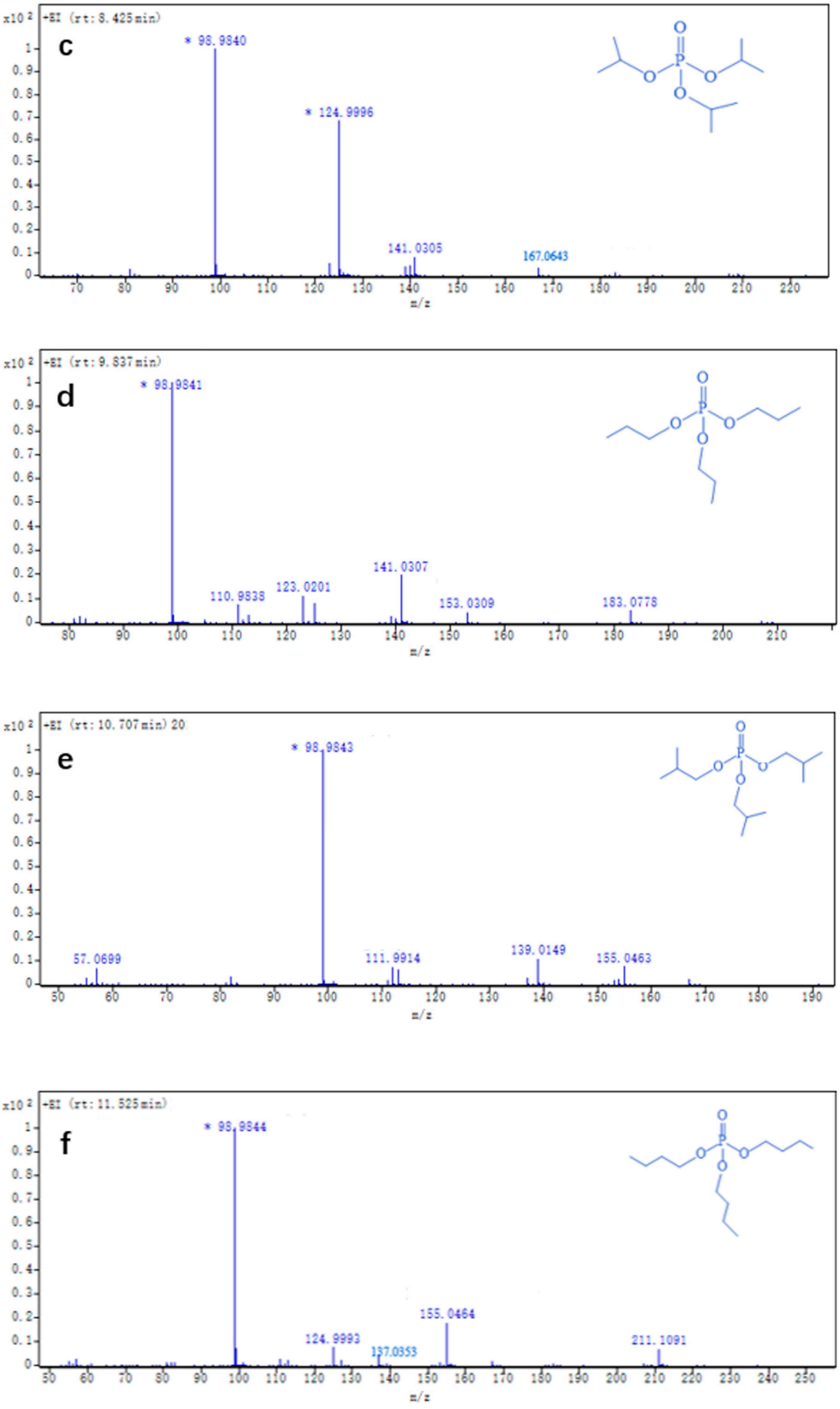
c
+EI (rt: 8.425min)
* 98.9840
* 124.9996
141.0305
167.0643
m/z
d
+EI (rt: 9.837min)
* 98.9841
110.9838
123.0201
141.0307
153.0309
183.0778
m/z
e
+EI (rt: 10.707min) 20
* 98.9843
57.0699
111.9914
139.0149
155.0463
m/z
f
+EI (rt: 11.525min)
* 98.9844
124.9993
137.0353
155.0464
211.1091
m/z

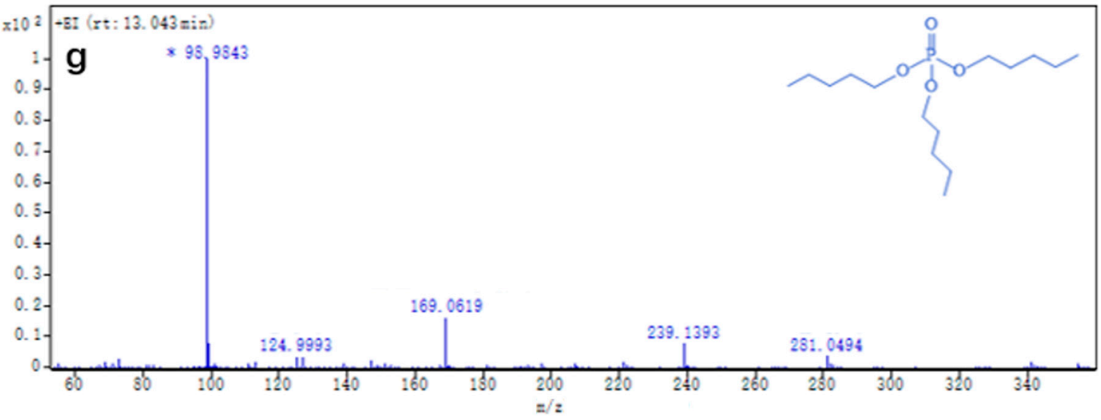
x10 2
+EI (rt: 13.043min)
g
* 98.9843
124.9993
169.0619
239.1393
281.0494
m/z

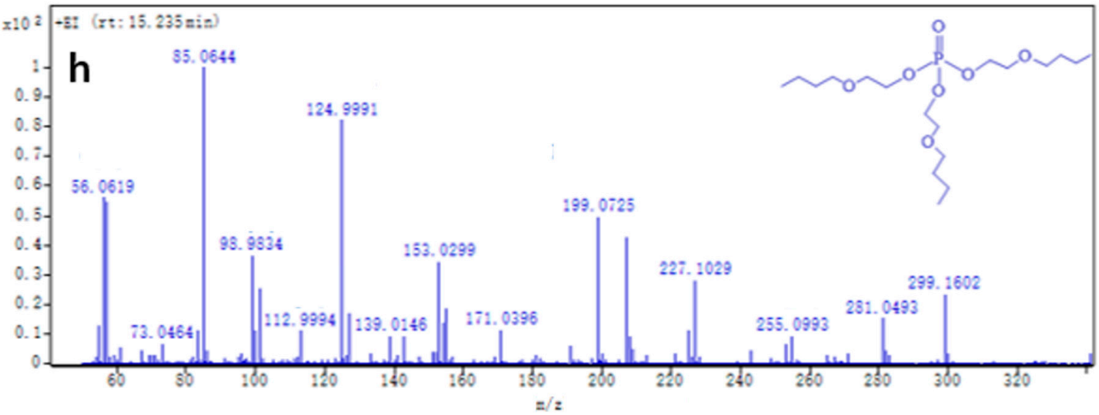
x10 2
+EI (rt: 15.235min)
h
85.0644
124.9991
56.0619
98.9834
73.0464
112.9994
139.0146
153.0299
171.0396
199.0725
227.1029
255.0993
281.0493
299.1602
m/z

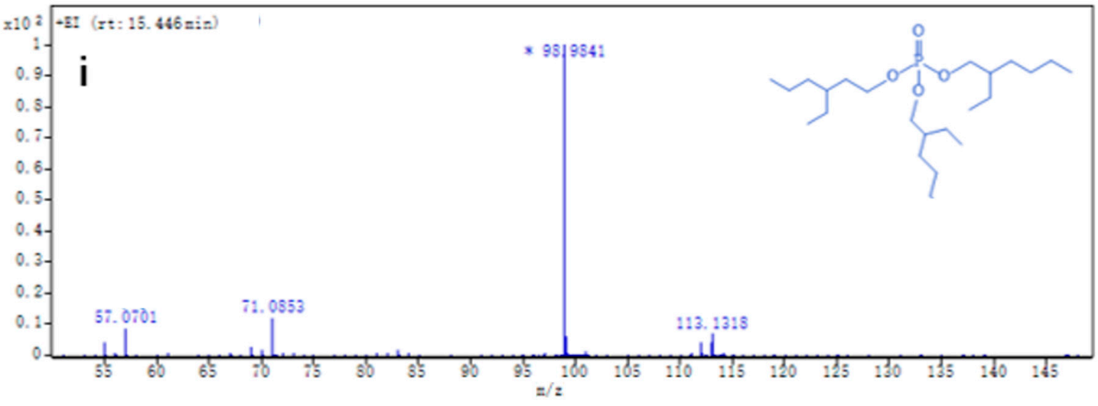
x10 2
+EI (rt: 15.446min)
i
* 98.9841
57.0701
71.0853
113.1318
m/z

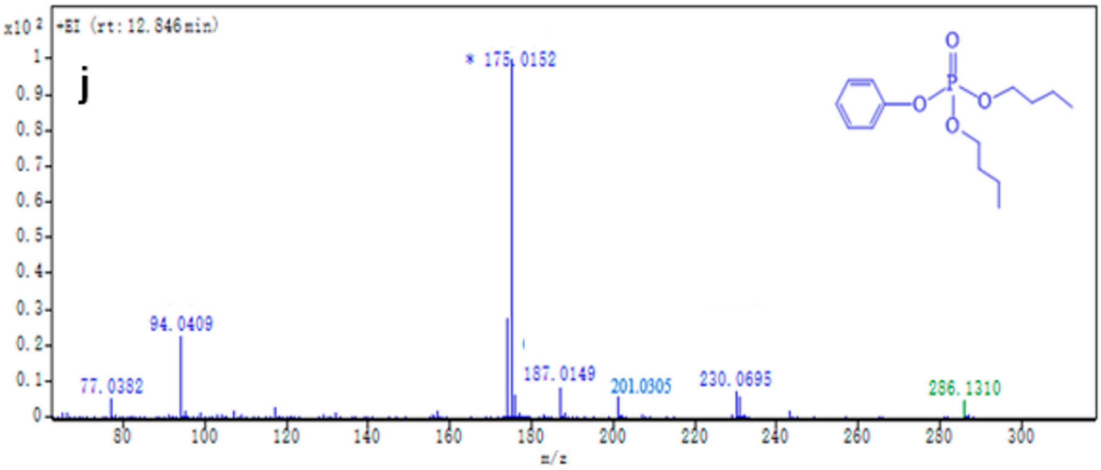
x10 2
+EI (rt: 12.846 min)
j
* 175.0152
94.0409
77.0382
187.0149
201.0305
230.0695
286.1310
m/z

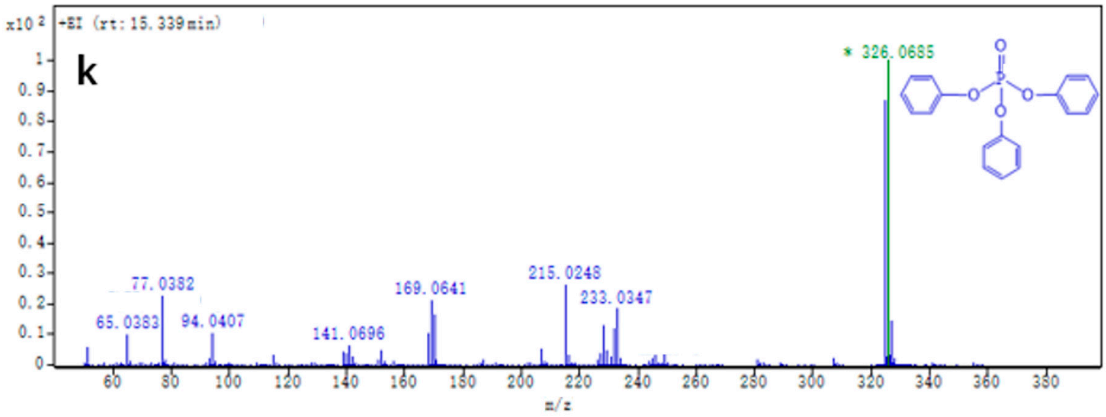
x10 2
+EI (rt: 15.339 min)
k
* 326.0685
77.0382
65.0383
94.0407
141.0696
169.0641
215.0248
233.0347
m/z

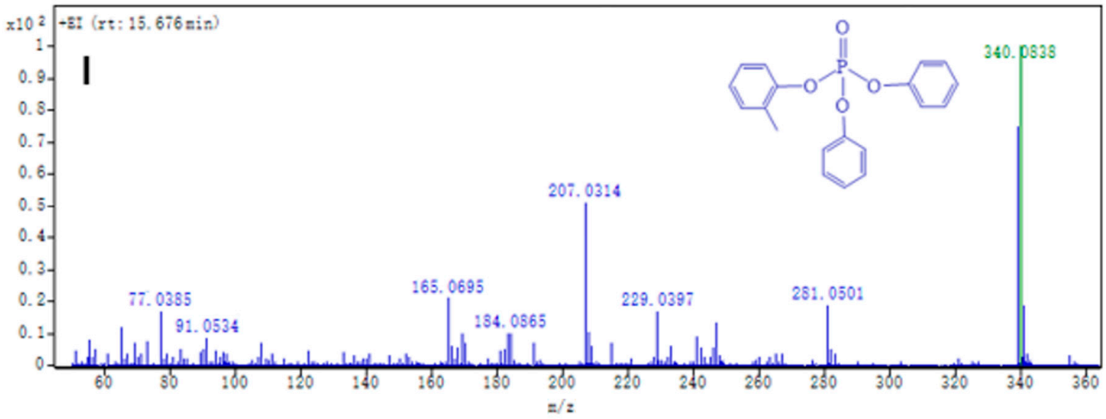
x10 2
+EI (rt: 15.676 min)
l
340.0838
207.0314
77.0385
91.0534
165.0695
184.0865
229.0397
281.0501
m/z

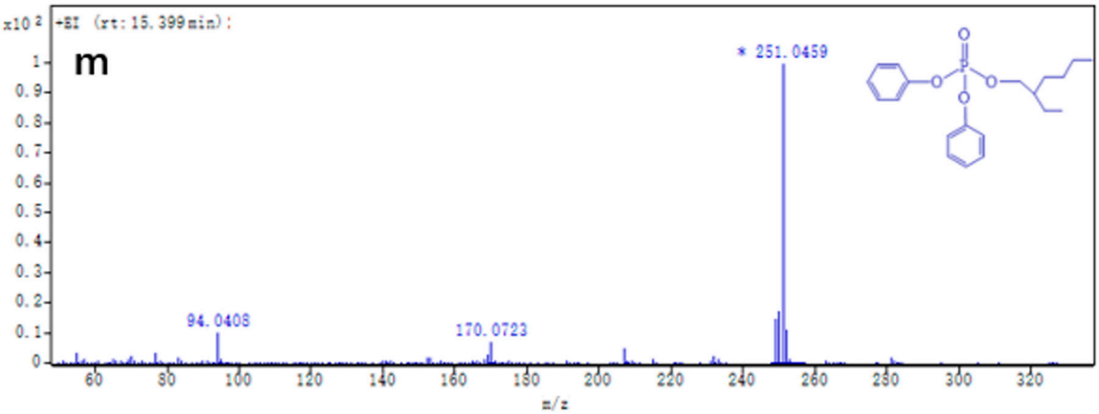
x10 2
+EI (rt: 15.399min):
m
* 251.0459
94.0408
170.0723
m/z

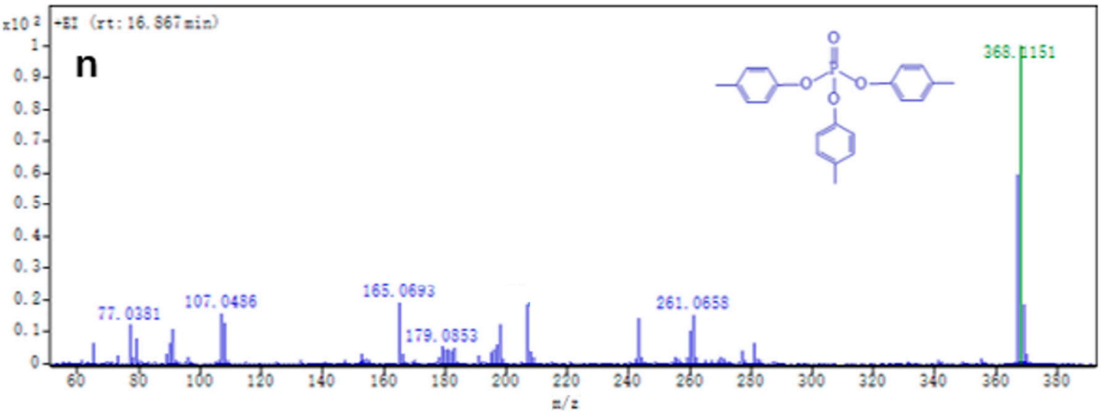
x10 2
+EI (rt: 16.867min)
n
368.1151
77.0381
107.0486
165.0693
179.0853
261.0658
m/z

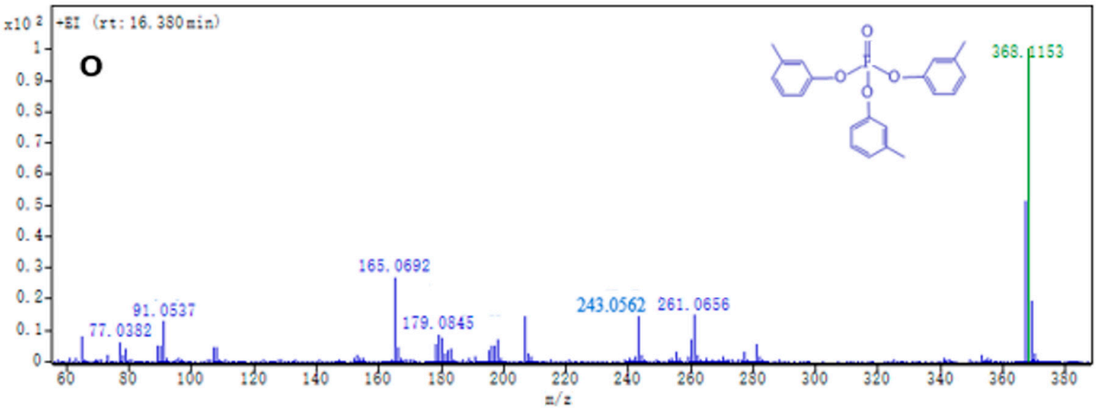
x10 2
+EI (rt: 16.380min)
o
368.1153
77.0382
91.0537
165.0692
179.0845
243.0562
261.0656
m/z

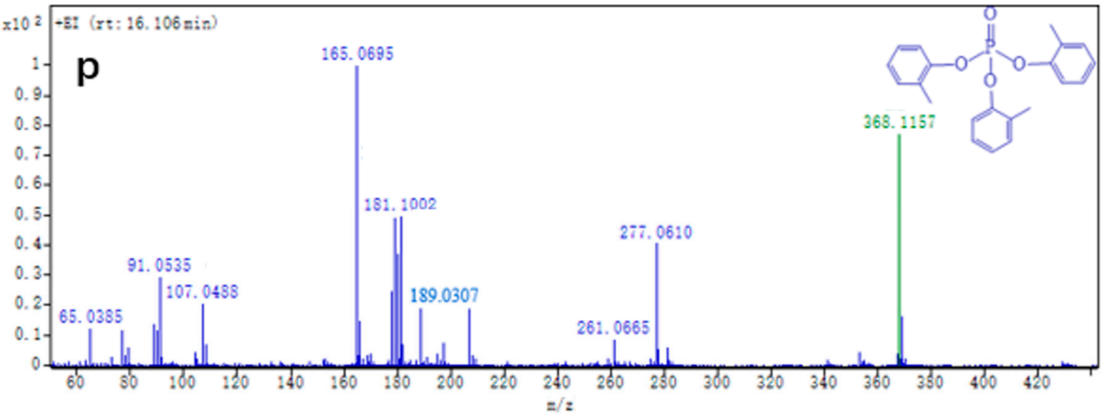

p
x10 2
+EI (rt: 16.106min)
165.0695
368.1157
181.1002
277.0610
91.0535
107.0488
189.0307
65.0385
261.0665
m/z

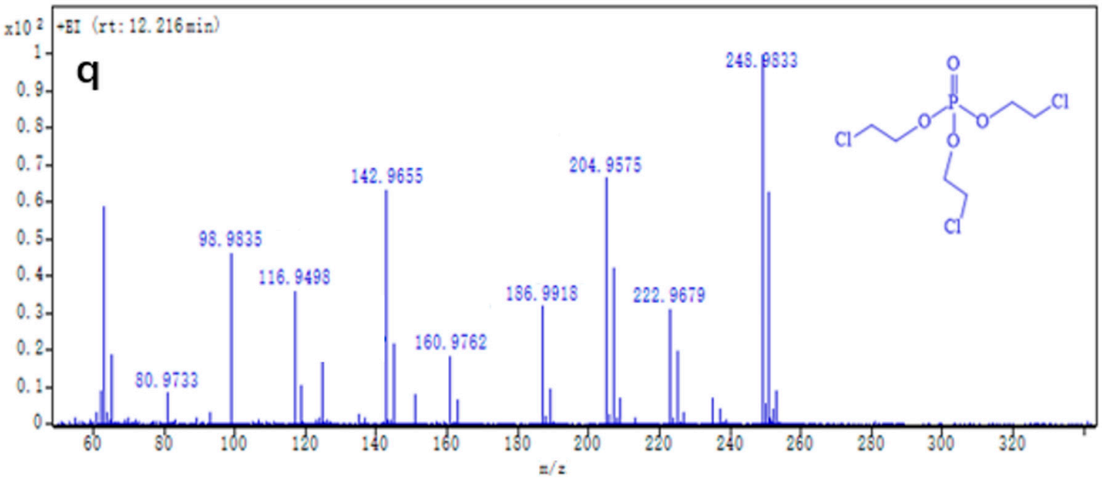

q
x10 2
+EI (rt: 12.216min)
248.9833
142.9655
204.9575
98.9835
116.9498
186.9918
222.9679
160.9762
80.9733
m/z

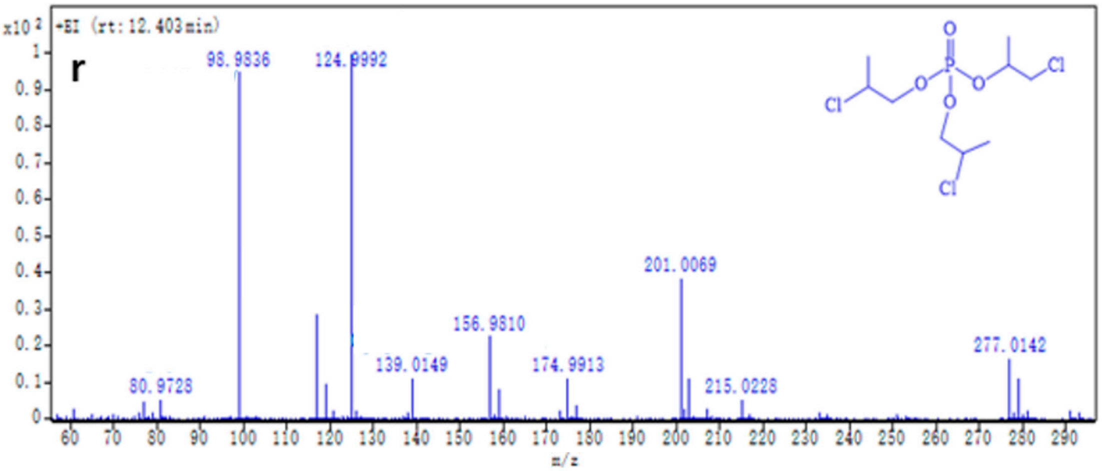

r
x10 2
+EI (rt: 12.403min)
98.9836
124.9992
201.0069
156.9810
139.0149
174.9913
277.0142
80.9728
215.0228
m/z

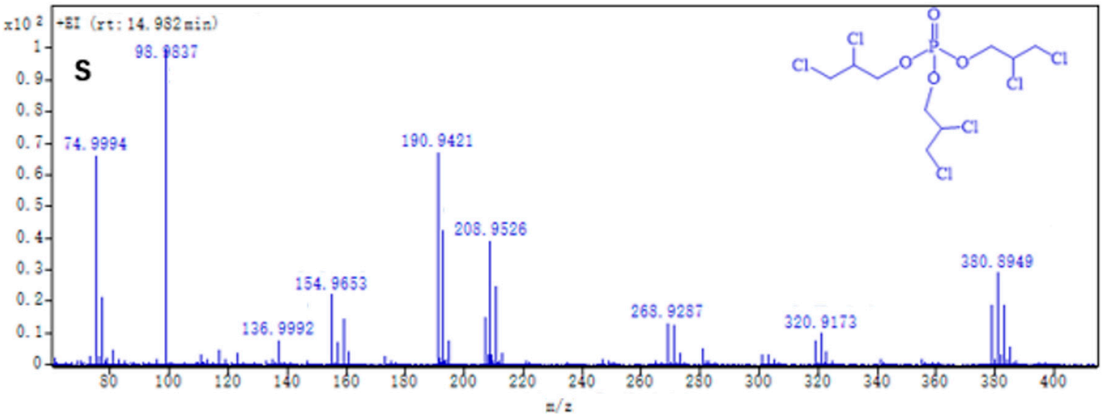

Figure S6. MS^2 spectrums of OPFRs under EI source

a. TMP; b. TEP; c. TiPP; d. TnPP; e. TiBP; f. TnBP; g. TPeP; h. TBOEP; i. TEHP; j. dBPhP; k. TPHP; l. CDPP; m. EHDPP; n. p-TCP; o. m-TCP; p. o-TCP; q. TCEP; r. TCIPP; s. T23DCIPP.

References

1. Yang, J. S.; Zhang, Q. H.; Su, L. Q. Fragmentation mechanism of organic phosphorus flame retardant by gas chromatography-quadrupole time of flight mass spectrometry. *J. Mass. Spectrom*, 2019, *42*, 36-47. [Google Scholar]
